# AKH-FOXO pathway regulates starvation-induced sleep loss through remodeling of the small ventral lateral neuron dorsal projections

**DOI:** 10.1371/journal.pgen.1009181

**Published:** 2020-10-26

**Authors:** Qiankun He, Juan Du, Liya Wei, Zhangwu Zhao

**Affiliations:** 1 Department of Entomology and MOA Key Lab of Pest Monitoring and Green Management, College of Plant Protection, China Agricultural University, Beijing, China; 2 College of Life Science, Hebei University, Baoding, China; Katholieke Universiteit Leuven, BELGIUM

## Abstract

Starvation caused by adverse feeding stresses or food shortages has been reported to result in sleep loss in animals. However, how the starvation signal interacts with the central nervous system is still unknown. Here, the adipokinetic hormone (AKH)—Fork head Box-O (FOXO) pathway is shown to respond to energy change and adjust the sleep of *Drosophila* through remodeling of the s-LNv (small ventral lateral neurons) dorsal projections. Our results show that starvation prevents flies from going to sleep after the first light-dark transition. The LNvs are required for starvation-induced sleep loss through extension of the pigment dispersing factor (PDF)-containing s-LNv dorsal projections. Further studies reveal that loss of AKH or AKHR (akh receptor) function blocks starvation-induced extension of s-LNv dorsal projections and rescues sleep suppression during food deprivation. FOXO, which has been reported to regulate synapse plasticity of neurons, acts as starvation response factor downstream of AKH, and down regulation of FOXO level considerably alleviates the influence of starvation on s-LNv dorsal projections and sleep. Taking together, our results outline the transduction pathways between starvation signal and sleep, and reveal a novel functional site for sleep regulation.

## Author summary

Sleep of *Drosophila* is regulated by circadian rhythm and homeostasis on the normal condition, in the presence of cellular stress or injury, the central nervous system neurons adjust sleep on the basis of impulse, which is transmitted by neuropeptides generated by cytokines from either peripheral tissues or nervous system cells. Metabolism and sleep are inextricably linked, energy deficiency has an enormous impact on the sleep, starvation induced sleep loss in the *Drosophila* has been reported and many genes play roles in this process. However, how the starvation signals transmit to nervous system and then make the sleep loss are still no clear. In this study we find the starvation prevent flies going to sleep, PDF/PDFR and LNvs are required for starvation induced sleep loss, during starvation flies show more expanded s-LNvs dorsal projection compared to flies on the normal condition. LNvs are vital awakening neurons, they transmit arousal signal to downstream neurons through its dorsal projections, more patulous s-LNvs dorsal projections accelerate the transmission of awakening signals, can prevent flies from going to sleep. Flies losing AKH/AKHR/FOXO function limit starvation induced sleep loss. Flies with blocked AKH-FOXO pathway also limit starvation induced s-LNvs dorsal projections opening, indicating that the s-LNvs dorsal projections are function sites of starvation signal on sleep regulation.

## Introduction

Starvation, a frequent result of feeding stresses or food shortages for animals, is not only a passively endured experience, but also a trigger for a reorganization of metabolism and behavior [1, 2]. The essence of starvation is that food intake does not meet energy needs. In response to energy deficiency, the organisms metabolize stored energy to meet the needs and drive a complex behavioral program to forage and ingest food.

In *Drosophila*, energy homeostasis is controlled by insulin like peptides (DILPs) and AKH. During feeding, DILPs cause class IIa deacetylase (HDAC4) to be phosphorylated and detained in the cytoplasm by upregulating the activity of Ser/Thr kinase 3 (SIK3), which prevents FOXO deacetylation and suppresses catabolic gene expression [3]. Under fasting condition, AKH enhances cAMP signaling by triggering the AKHR in the fat body [4]. The AKH/AKHR pathway cause a SIK3 phosphorylation decrease, which leads to the dephosphorylation and nuclear translocation of HDAC4 and FOXO deacetylation, and consequently induces the expression of *brummer* to break down stored lipids for energy [5].

In addition to the studies on starvation-induced metabolism shifts, starvation-induced sleep loss and hyperactivity in flies also have attracted attention. AKH was first found to function in starvation-induced locomotor activity in 2004, when studies showed that AKH-cell-deficient (AKH-CD) flies are resistant to starvation-induced death and hyperactivity. It was concluded that AKH as a metabolism stimulator functions to mobilize the stored energy for maximizing flies’ survival when food is scarce [6]. Since the finding of starvation-induced sleep loss and hyperactivity, a number of effective factors were identified in *Drosophila* [7, 8], in which *clock* and *cycle* inhibit starvation-induced sleep loss [7]. Additionally, octopamine [8], AKHR [9], translin [10], neuropeptide F (NPF) [11], serine [12] and leucokinin (Lk) [13] are also required for starvation-mediated behavioral changes.

Sleep and feeding are conflicting behaviors, and their time sequences are anticipated by circadian rhythms in synchrony with external cues. Sleep has an obvious characteristic of periodic changes regulated by the circadian rhythm and intrinsic homeostatic systems [14], in which the central neuronal circuits account for sleep regulation [15, 16]. Clock neurons, mushroom bodies (MB), the central complex and the Pars Intercerebralis (PI) have been shown to regulate the sleep-wake cycle. Clock and cycle limit starvation-induced sleep loss, which means that clock neurons play an important role in deciding the time of sleep or food search during starvation [7, 17]. Clock neurons, mainly including LNvs, LNds (dorsal lateral neurons) and DN1s (dorsal neurons), form a feedback loop to control sleep-activity of Drosophila [18]. The PDF-positive l-LNvs and s-LNvs (M cells) are known as arousal neurons. Loss of PDF-neurons or PDF itself increases the amount of daytime sleep [19, 20], while the CRY-positive LNds and the 5th s-LNv (E cells) control the amount of nighttime sleep; activation of the E cells causes sleep loss [20–22]. M cells activate the DN1s to promote awakening, while DN1-mediated inhibitory feedback on M and E cells promotes the siesta and night-time sleep [18, 22–24]. Both s-LNvs and LNds directly connect to the DN1s through the projections in the dorsal protocerebrum. Additionally, PDF neurons also modulate the phase of E cell oscillations [18, 22]. PDF containing s-LNv dorsal projections exhibit a clock-controlled structural plasticity [25], in which genes and microRNAs such as *period*(*per*) and *timeless*(*tim*) [25], Histone Acetyltransferase Tip60 (HAT *Tip60*) [26], Myocyte enhancer factor 2 (*Mef2*) [27], microRNA-92a [28], and microRNA-263b [29] involved in s-LNvs axonal fasciculation have been shown to impact circadian behavior. These data imply s-LNvs dorsal projection remodeling is required to propagate the time of information from core pacemaker cells to downstream targets for underlying rhythmic behavior.

*Drosophila* sleep and activity are controlled by a complex neural network. To determine how the starvation signal is transmitted to the central nervous system and then regulates sleep, we first studied the influence of starvation on sleep, and then clarified the regulatory mechanism of starvation-induced sleep loss.

## Results

### Starvation prevents flies from going to sleep

**Fig 1:**
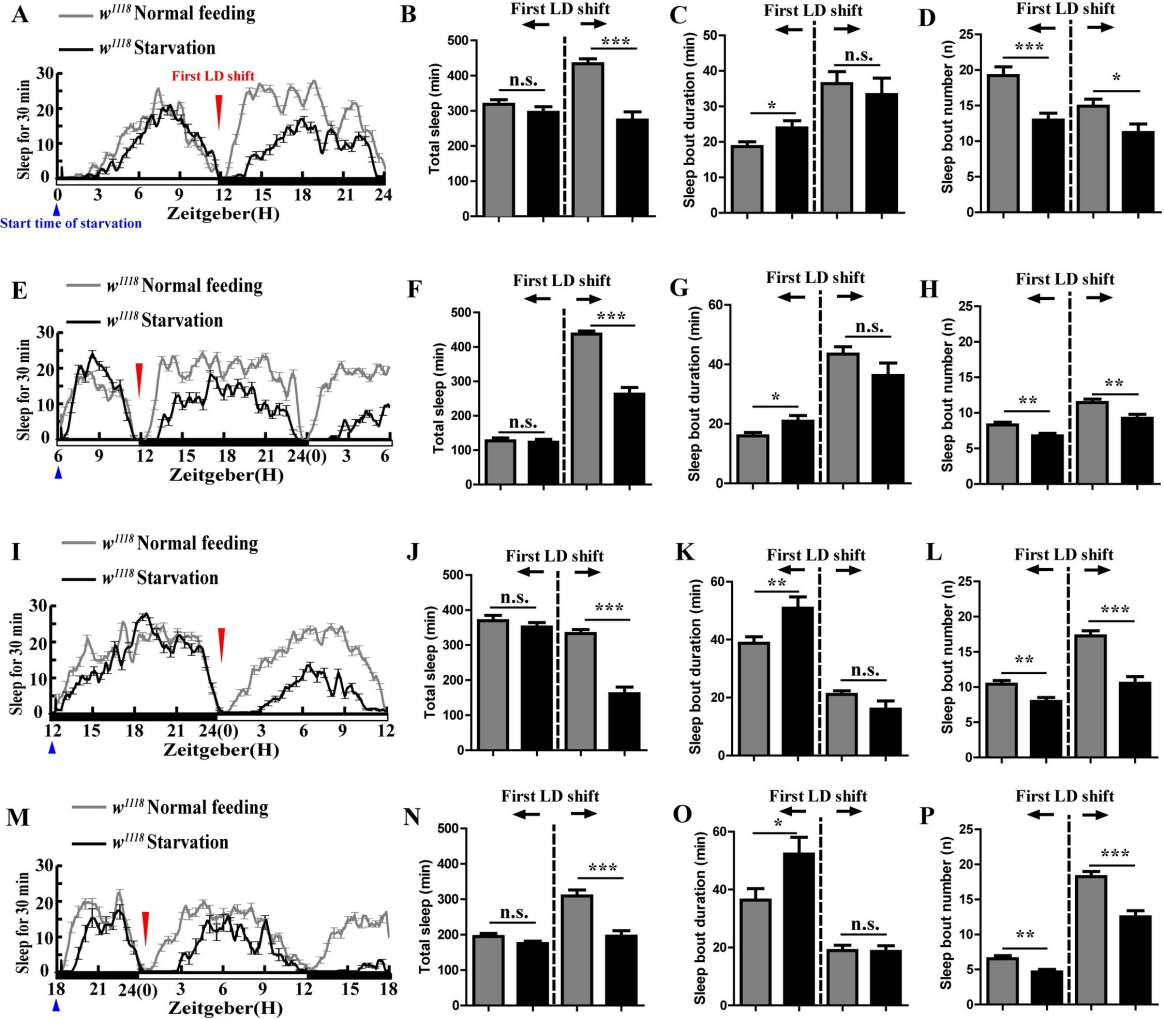
Starvation prevents the Drosophila from going to sleep. (A) Sleep profile of wild type flies (*w^1118^*) under normal condition (gray line) (n = 43) and starvation (black line) (n = 31). The blue triangle indicates start time of food deprivation (ZT0). The red inverted triangle indicates the first light-dark (LD) shift during starvation. The white bar indicates light period, the black bar indicates the dark phase. (B to D) Total sleep (B), sleep bout duration (C), and sleep bout number (D) of *w^1118^* flies before and after first LD shift during normal feeding (n = 43) and starvation starting at ZT0 (n = 31). Gray column indicates the data of normal feeding, black column indicates the data of starvation. The data were analyzed by t test, ***p<0.0001 and *p< 0.05. (E) Sleep profile of *w^1118^* under normal condition (gray line) (n = 65) and starvation (black line) (n = 60). The blue triangle indicates start time of food deprivation (ZT6). The red inverted triangle indicates the first light-dark (LD) shift during starvation. The white bar indicates light period, the black bar indicates the dark phase. (F to H) Total sleep (F), sleep bout duration (G), and sleep bout number (H) of *w^1118^* flies before and after first LD shift during normal feeding (n = 65) and starvation starting at ZT6 (n = 60). Gray column indicates the data of normal feeding, black column indicates the data of starvation. The data were analyzed by t test, ***p<0.0001, **p<0.001 and *p< 0.05. (I) Sleep profile of *w^1118^* under normal condition (gray line) (n = 44) and starvation (black line) (n = 38). The blue triangle indicates start time of food deprivation (ZT12). The red inverted triangle indicates the first light-dark (LD) shift during starvation. The white bar indicates light period, the black bar indicates the dark phase. (J to L) Total sleep (J), sleep bout duration (K), and sleep bout number (L) of *w^1118^* flies before and after first LD shift during normal feeding (n = 44) and starvation starting at ZT12 (n = 38). Gray column indicates the data of normal feeding, black column indicates the data of starvation. The data were analyzed by t test, ***p<0.0001 and **p<0.001. (M) Sleep profile of *w^1118^* under normal condition (gray line) (n = 42) and starvation (black line) (n = 52). The blue triangle indicates start time of food deprivation (ZT18). The red inverted triangle indicates the first light-dark (LD) shift during starvation. The white bar indicates light period, the black bar indicates the dark phase. (N to P) Total sleep (N), sleep bout duration (O), and sleep bout number (P) of *w^1118^* flies before and after first LD shift during normal feeding (n = 42) and starvation starting at ZT18 (n = 52). Gray column indicates the data of normal feeding, black column indicates the data of starvation. The data were analyzed by t test, ***p<0.0001, **p<0.001 and *p< 0.05.

Previous studies have shown that sleep is significantly suppressed over 12–24 hours of starvation, whether the time of starting starvation is at ZT0 or ZT12 [7]. In order to further clarify the role and mechanism of starvation-induced sleep loss, we studied sleep behavior of the wild-type (*w*^*1118*^) flies over time from the start of starvation until 24 h of starvation under a 12 h L (light):12 h D (dark) condition, in which ZT0 and ZT12 represent lights on and lights off (respectively), and starvation started at ZT0, ZT6, ZT12 and ZT18. Results showed that starvation-induced sleep loss began from the period of light/dark shift, independent from the time at which the flies were starved (Fig 1A, 1E, 1I and 1M). These data indicate that starvation-induced sleep loss starts at a light/dark shift that is either from light to dark or from dark to light.

Moreover, the starvation-induced sleep loss, during 12 hours after a light/dark shift, is mainly dependent on a decrease of the sleep bout number and is not related to the sleep bout duration (Fig 1B–1D, 1F–1H, 1J–1L and 1N–1P). Only reduction of the sleep bout number should respond to a condition in which it is more difficult to fall asleep. These results illustrate that the starvation-induced sleep loss is due to a condition causing flies to have more difficulty to fall asleep.

### PDF and its neurons are involved in starvation-induced sleep loss

**Fig 2:**
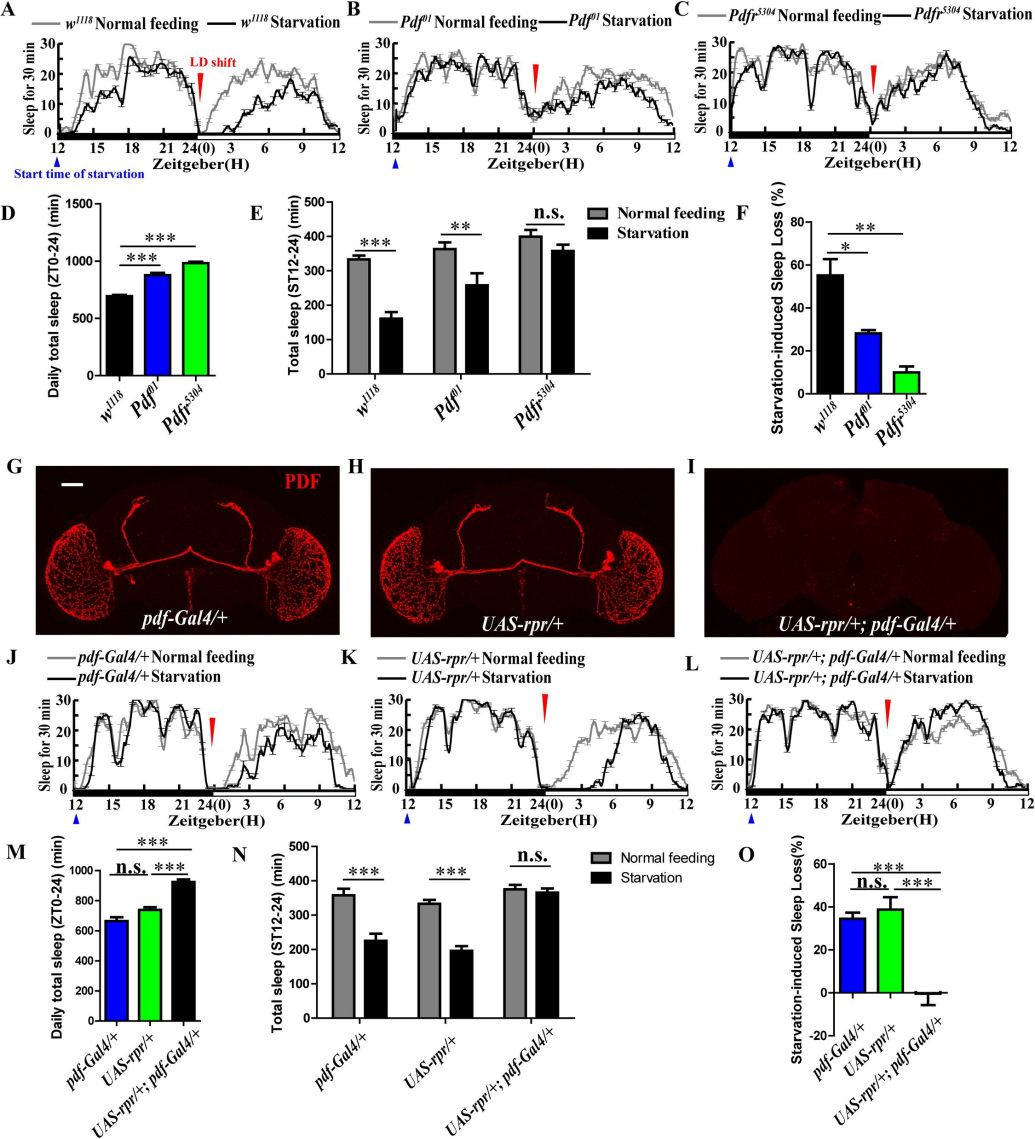
PDF and PDF neurons are required for starvation-induced sleep loss. (A to C) Sleep profile of *w^1118^* (A), *Pdf^01^* (B), and *Pdfr^5304^* (C) under normal condition (gray line) and starvation (black line). The blue triangle indicates start time of food deprivation (ZT12). The red inverted triangle indicates the first light-dark (LD) shift during starvation. (D) Daily total sleep of *w^1118^* (black column), *Pdf^01^* (blue column), and *Pdfr^5304^* (green column) flies. Data were analyzed by One-way ANOVA, Dunnett’s Multiple Comparison Test. ***p<0.0001. (E) Sleep time of *w^1118^*, *Pdf^01^*and *Pdfr^5304^* flies during ST12-St24 (Starvation time 12–24; ZT0-ZT12) under normal feeding condition (gray columns) and starvation (black columns). Data were analyzed by t test, ***p<0.0001 and **p<0.001. (F) Starvation induced sleep loss in *w^1118^* (black column), *Pdf^01^* (blue column), and *Pdfr^5304^* (green column) flies. Data were analyzed by One-way ANOVA, Dunnett’s Multiple Comparison Test. **p<0.001 and *p< 0.05. (G to I) Anti-PDF immunostaining in *pdf-Gal4/+* (G), *UAS-rpr/+* (H), and *UAS-rpr/+*; *pdf-Gal4/+* (I) flies. (J to L) Sleep profile of *pdf-Gal4/+* (J), *UAS-rpr/+* (K), and *UAS-rpr/+*; *pdf-Gal4/+* (L) under normal condition (gray line) and starvation (black line). The blue triangle indicates start time of food deprivation (ZT12). The red inverted triangle indicates the first light-dark (LD) shift during starvation. (M) Daily total sleep of *pdf-Gal4/+* (blue column), *UAS-rpr/+* (green column), and *UAS-rpr/+*; *pdf-Gal4/+* (black column) flies. Data were analyzed by One-way ANOVA, Tukey’s Multiple Comparison Test, ***p<0.0001. (N) Sleep time of *pdf-Gal4/+*, *UAS-rpr/+* and *UAS-rpr/+*; *pdf-Gal4/+* flies during ST12-ST24 (ZT0-ZT12) under normal feeding condition (gray columns) and starvation (black columns). Data were analyzed by t test, ***p<0.0001 and **p<0.001. (O) Starvation-induced sleep loss in *pdf-Gal4/+* (blue column), *UAS-rpr/+* (green column), and *UAS-rpr/+*; *pdf-Gal4/+* (black column) flies. Data were analyzed by One-way ANOVA, Tukey’s Multiple Comparison Test, **p<0.001 and *p< 0.05.

In order to determine whether the starvation-induced sleep loss is related to the neuropeptide PDF, an important factor of sleep regulation, we monitored sleep behavior in *Pdf* ^*01*^ and *Pdfr*^*5304*^ mutant flies during normal feeding and after 24 h of food deprivation, respectively (same background of *w*^*1118*^ as control) (Fig 2A–2C). Results showed that *Pdf* ^*01*^ and *Pdfr*^*5304*^ mutant flies sleep more than *w*^*1118*^ under non-starvation conditions (Fig 2D). *Pdf* ^*01*^ may partially but the *Pdfr*^*5304*^ may almost completely resist the starvation-induced sleep loss compared to *w*^*1118*^ control flies (Fig 2A–2C and 2E), in which the rate of starvation-induced sleep loss in the *Pdf* ^*01*^ and *Pdfr*^*5304*^ flies is significantly reduced to 28.12% and 9.94% respectively compared to 55.15% in *w*^*1118*^ (Fig 2F).

Moreover, we used the *pdf-Gal4* to drive the expression of a cell death gene (*UAS-rpr*) in the PDF clock neurons, which causes the PDF neurons and its projections to be absent in brains of adult flies (Fig 2G–2I). Then, we monitored sleep behaviors under normal feeding and after 24 h of food deprivation among flies with PDF neurons ablated (*UAS-rpr/+; pdf-Gal4*) and the same background of controls (*pdf-Gal4/+* and *UAS-rpr/+*). Results showed the same sleep phenotype, like the *Pdf* ^*01*^ and *Pdfr*^*5304*^ flies, with increased daily total sleep (Fig 2M). The flies with missing PDF neurons completely resist the starvation-induced sleep loss compared to those in controls (Fig 2J–2L and 2N), in which the rate of starvation-induced sleep loss in the treatment flies is significantly reduced to -0.36% compared to 34.45% in the *pdf-Gal4/+* and 38.73% in the *UAS-rpr/+* controls (Fig 2O). Taking together, these results indicate that PDF and PDF neurons play an important role on starvation-induced sleep loss.

### Starvation induces extension of s-LNv dorsal projections

**Fig 3:**
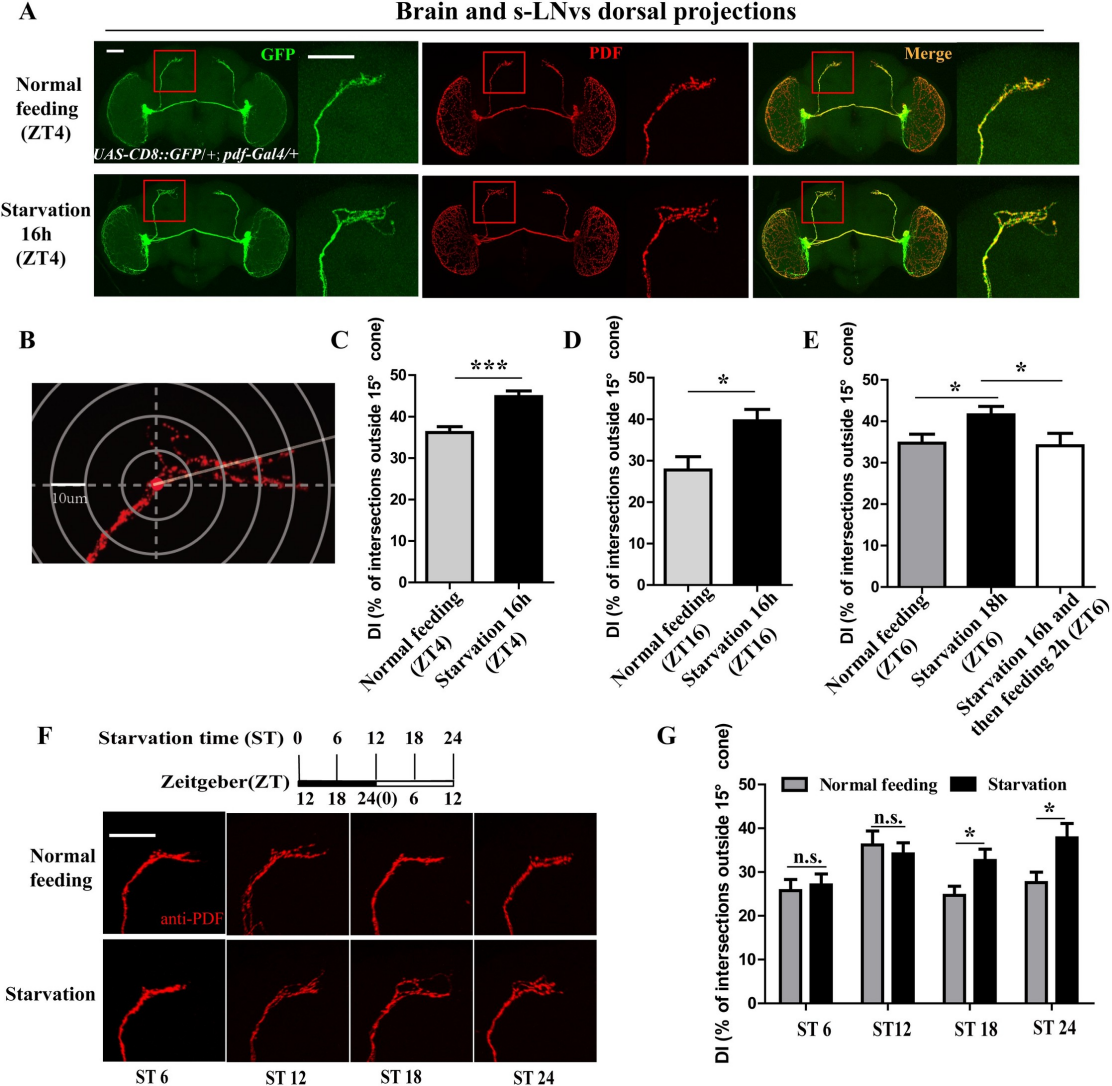
Starvation-induced extension of s-LNv dorsal projections. (A) The immunostaining of *UAS-CD8::GFP/+*; *pdf-Gal4/+* flies under normal feeding condition at ZT4 and starvation condition at ST16 (ZT4) with anti-GFP (green) and anti-PDF (red). The scale bar indicates 50um. (B) The diagram of quantification of fasciculation of s-LNv dorsal projections. Defasciculation index (DI), percentage of intersections between concentric rings and dorsal projections outside of a 15% cone, was introduced to indicate the extent of synaptic diffusion (refer to 27). (C) DI of *UAS-CD8::GFP/+*; *pdf-Gal4/+* flies under normal condition at ZT4 (gray columns) and during starvation at ST16 (ZT4) (black column). The DI’s were calculated using PDF immunofluorescence (which completely overlaps GFP immunofluorescence in s-LNv dorsal projections in *UAS-CD8::GFP/+*; *pdf-Gal4/+* flies). Data were analyzed by t test, ***p<0.0001. (D) DI of *UAS-CD8::GFP/+*; *pdf-Gal4/+* flies under normal condition at ZT16 (gray columns) and during starvation at ST16 (ZT16) (black column). Data were analyzed by t test, *p< 0.05. (E) DI of *w^1118^* flies under normal condition at ZT6 (gray columns), during starvation at ST18 (ZT6) (black column) and during food deprivation for 16h and then refeeding 2h (ZT6) (white column). Data were analyzed by t test, *p< 0.05. (F) PDF staining in *w^1118^* flies during starvation at ST6 (ZT18), ST12 (ZT0), ST18 (ZT6), ST24 (ZT12) and flies under normal feeding at same points. (G) DIs of *w^1118^* flies under normal feeding condition (gray column) and food deprivation (black column) at ST6 (ZT18), ST12 (ZT0), ST18 (ZT6), ST24 (ZT12). Data were analyzed by t test, *p< 0.05.

To confirm the mechanism of PDF and its neurons on starvation-induced sleep loss, we constituted a *w*^*1118*^ background of fly line with PDF neuron-specific membrane-tethered GFP (mCD8::GFP), a stable reporter for analysis of the PDF-expressing neurons and projections. By analyzing the fly brain of this line with PDF antibodies, the PDF neurons at ZT4 were detected in both flies after 16 h starvation and without starvation, because ZT4 showed significant starvation-induced sleep loss (Fig 2J–2L). Results showed that the s-LNvs dorsal projections in starved flies are more open compared with those in non-starved flies (Fig 3A and 3B). With a previously described method for analysis of s-LNv dorsal projections [27], we found that the s-LNv dorsal termini defasciculation index (DI, percentage of intersections between concentric rings and axonal branches outside of a 15% cone) detected at both ZT4 and ZT16 in the starved 16 h flies was significantly increased compared to those in non-starved control (Fig 3C and 3D). However, PDF intensity in LNv somas or dorsal projections showed no significant difference between starved and non-starved flies (S1 Fig). These results indicated that food deprivation induces the s-LNv dorsal projections remodeling.

To further confirm the role of starvation on s-LNv dorsal projection remodeling, we detected the s-LNv dorsal projections of flies that were subjected to food deprivation for 16 h and then refed for 2 h; the results showed that refeeding could offset the expanded s-LNv dorsal projections in the flies with sustained starvation (Fig 3E). In order to understand when the s-LNv dorsal projections remodeling occurs after starvation, we further measured the states of the s-LNvs dorsal projections in *w*^*1118*^ flies at every 6 h of starvation until 24 h. Results showed that the s-LNv dorsal termini became more expansive in flies after 18 h and 24 h of starvation as compared to those in non-starved flies (Fig 3F and 3G), the time when the s-LNv dorsal projections changed is response to behaviors of starvation induced sleep loss occurred after first LD shift. These results indicate that hunger stimulation is one of the regulatory factors of the s-LNv dorsal projection remodeling, and the s-LNv dorsal projection remodeling accounts for the starvation-induced sleep loss after LD shift.

### AKH is indispensable for starvation-induced sleep loss and extension of PDF-containing s-LNv dorsal projections

**Fig 4:**
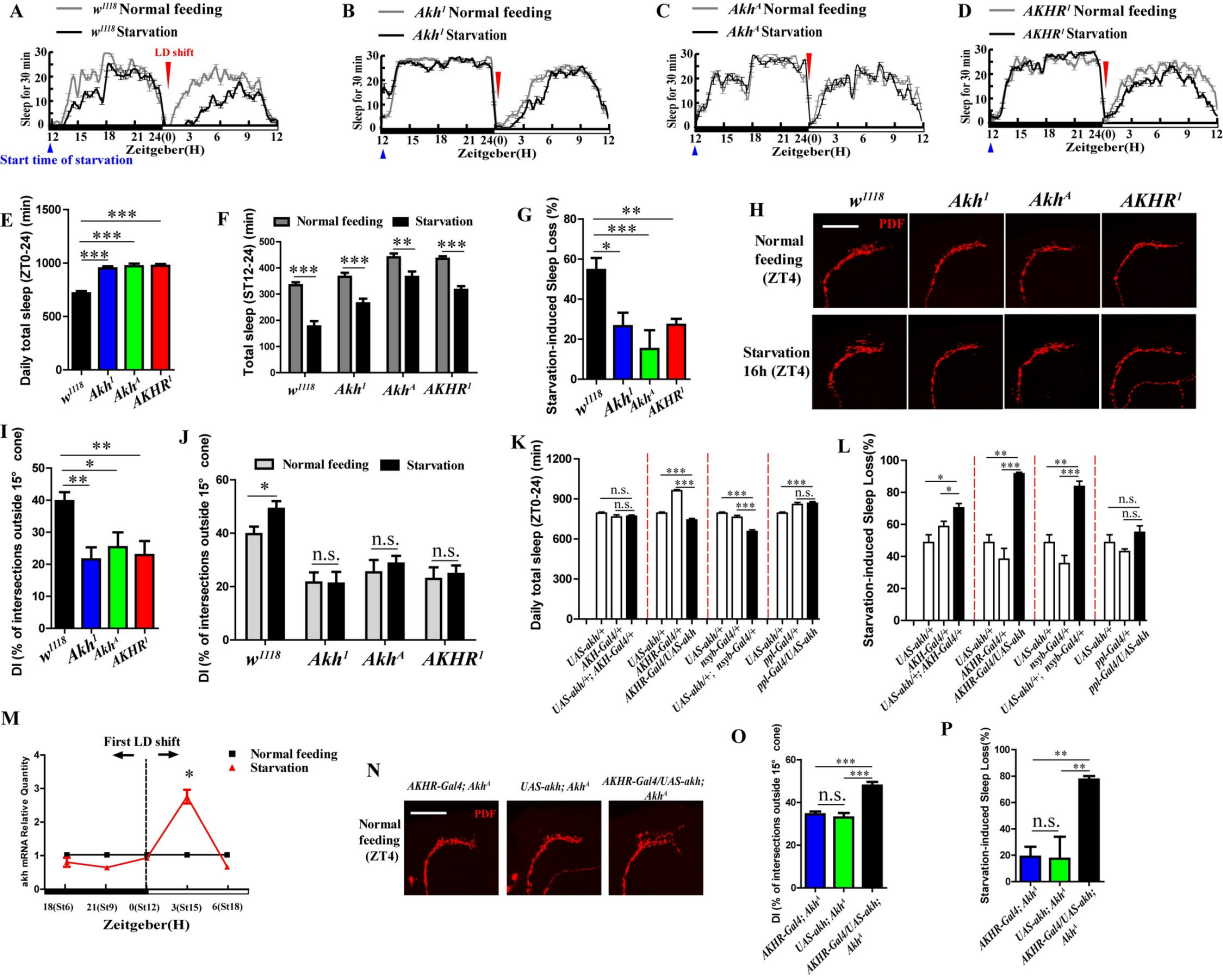
AKH is indispensable for starvation-induced sleep loss and extension of PDF-containing s-LNv dorsal projections. (A to D) Sleep profile of *w^1118^* (A), *Akh^1^* (B), *Akh^A^* (C), and *AKHR^1^* (D) flies under normal condition (gray line) and starvation (black line). The blue triangle indicates start time of food deprivation (ZT12). The red inverted triangle indicates the first light-dark (LD) shift during starvation. (E) Daily total sleep of *w^1118^* (black column), *Akh^1^* (blue column), *Akh^A^* (green column), and *AKHR^1^* (red column) flies. Data were analyzed by One-way ANOVA, Dunnett’s Multiple Comparison Test. ***p<0.0001. (F) Sleep time of *w^1118^*, *Akh^1^*, *Akh^A^*, and *AKHR^1^* flies during ST12-ST24 (ZT0-ZT12) under normal feeding condition (gray columns) and starvation (black columns). Data were analyzed by t test, ***p<0.0001 and **p<0.001. (G) Starvation induced sleep loss in *w^1118^* (black column), *Akh^1^* (blue column), *Akh^A^* (green column), and *AKHR^1^* (red column) flies. Data were analyzed by One-way ANOVA, Dunnett’s Multiple Comparison Test. ***p<0.0001, **p<0.001 and *p< 0.05. (H) PDF staining in *w^1118^*, *Akh^1^*, *Akh^A^*, and *AKHR^1^* flies under normal feeding condition at ZTt4 and starvation condition at ST16 (ZT4). The scale bar indicates 50um. (I) DIs of *w^1118^* (black column), *Akh^1^* (blue column), *Akh^A^* (green column), and *AKHR^1^* (red column) flies under normal feeding condition at ZT4. Data were analyzed by One-way ANOVA, Dunnett’s Multiple Comparison Test. **p<0.001 and *p< 0.05. (J) DI of *w^1118^*, *Akh^1^*, *Akh^A^*, and *AKHR^1^* flies under normal condition at ZT4 (gray column) and during starvation at ST16 (ZT4) (black column). Data were analyzed by t test, *p< 0.05. (K) Daily total sleep of *akh* overexpression flies driving by *AKH-Gal4*, *AKHR-Gal4*, *nsyb-Gal4*, and *ppl-Gal4* (black column) and its controls (white column). Data were analyzed by One-way ANOVA, Tukey’s Multiple Comparison Test. ***p<0.0001. (L) Starvation induced sleep loss in *akh* overexpression flies driving by *AKH-Gal4*, *AKHR-Gal4*, *nsyb-Gal4*, and *ppl-Gal4* (black column) and its controls (white column). Data were analyzed by One-way ANOVA, Tukey’s Multiple Comparison Test. ***p<0.0001, and *p< 0.05. (M) qRT-PCR analysis of *akh* amounts in *w^1118^* flies during starvation before and after first LD shift. Black cube indicates *akh* expression in the normal feeding condition, and the red triangle indicates the *akh* expression in the starvation condition (relative to its expression in the normal feeding). Data were analyzed by t test, *p< 0.05. (N) PDF staining in *AKHR-Gal4*; *Akh^A^*, *UAS-akh*; *Akh^A^* and *AKHR-Gal4/UAS-akh*; *Akh^A^* flies under normal feeding condition at ZT4 and starvation condition at ST16 (ZT4). The scale bar indicates 50um. (O) DIs of *AKHR-Gal4*; *Akh^A^* (blue column), *UAS-akh*; *Akh^A^* (green column), and *AKHR-Gal4/UAS-akh*; *Akh^A^* (red column) flies under normal feeding condition at ZT4. Data were analyzed by One-way ANOVA, Tukey’s Multiple Comparison Test. ***p<0.0001. (P) Starvation induced sleep loss in *AKHR-Gal4*; *Akh^A^* (blue column), *UAS-akh*; *Akh^A^* (green column), and *AKHR-Gal4/UAS-akh*; *Akh^A^* (black column) flies. Data were analyzed by One-way ANOVA, Tukey’s Multiple Comparison Test, **p<0.001.

To figure out the mechanism for the PDF-containing s-LNv dorsal projection extension, we focused on the AKH (adipokinetic hormone) pathway, which has been reported to be involved in starvation induced metabolism shifting and hyperactivity [5, 6]. We first monitored the sleep phenotypes of *Akh*^*1*^, *Akh*^*A*^, and *AKHR*^*1*^ (*AKHR* deletion) mutant flies under starvation and non-starvation conditions, in which the *Akh*^*1*^ loses function by missing the second amino acid (Leu) at the N-terminal end [30], and the *Akh*^*A*^ lacks the coding sequences of two amino acids (Asp and Trp) at the 3’-end [31]. Results showed that the mutant flies show a significant reduction in starvation-induced sleep loss of only 26.51%, 16.26 and 24.68% for the *Akh*^*1*^, *Akh*^*A*^, and *AKHR*^*1*^ flies respectively as compared to 52.52% in w1118 control flies (Fig 4A–4D and 4F and 4G). Under non-starvation conditions, these mutant flies also slept significantly longer than *w*^*1118*^ control flies (Fig 4E). These results indicate that AKH/AKHR are indispensable for starvation induced sleep loss.

To determine whether the AKH/AKHR pathway is associated with remodeling of the s-LNvs dorsal projections, we detected the PDF neurons of *w*^*1118*^ and AKH/AKHR mutant flies under both 16 h (ZT4) starved and non-starved conditions. Under the normal feeding condition, the DIs of *akh* or *AKHR* mutant flies were decreased compared with *w*^*1118*^ flies (Fig 4H and 4I). These data demonstrate that loss of *akh* or *AKHR* function blocks the extension of s-LNv dorsal projections, which corresponds to the increased sleep showed in *Akh*^*1*^, *Akh*^*A*^ and AKHR^1^ flies. During 16 h of starvation, we found that the DIs (at ZT4) in *w*^*1118*^ flies significantly increased in the starved flies compared with those in the non-starved flies (Fig 4J). However, the starvation-induced s-LNv dorsal projections extension disappeared in the *Akh*^*1*^, *Akh*^*A*^ or *AKHR*^*1*^ flies, in which DIs of these mutant flies were not significantly different under starvation and non-starvation conditions (Fig 4J). These data indicate that loss of *akh* or *AKHR* function blocks the starvation-induced s-LNv dorsal projections remodeling. Combined with resistances to the starvation-induced sleep loss in these flies, these results reveal that the s-LNv dorsal projection remodeling is related to the AKH/AKHR pathway.

Next, we overexpressed *akh* through the *UAS-akh* line driven by *AKH-Gal4* [specifically expressed in the corpora cardiaca (CC) and extended to the brain (S2 Fig)], *AKHR-Gal4* [expressed in fat body and some gustatory neurons (S3 Fig)], *nsyb-Gal4* [expressed in neurons not in fat body (S3 Fig)] and *ppl-Gal4* [expressed in fat body not in neurons (S3 Fig)] lines respectively to monitor sleep under non-starvation and starvation. The flies with *akh* overexpression driven by *AKHR-Gal4* and *nsyb-Gal4* decreased daily total sleep amount compared with their controls, but the *akh* overexpression driven in the *akh* and fat body cells did not show significant differences (Fig 4K). To further verify the effectiveness of *akh* overexpression, we measured trehalose levels of these *akh* overexpressed adult flies. The results showed that trehalose levels in *AKHR-Gal4/UAS-akh* and *UAS-akh/+*; *nsyb-Gal4/+* flies had significant increases, but not in *UAS-akh/+*; *AKH-Gal4/+* and *ppl-Gal4/UAS-akh* flies (S4 Fig). Different from *akh* overexpression in the fat body flies, *akh* overexpression flies driven by *AKH-Gal4*, *AKHR-Gal4* and *nsyb-Gal4* have more starvation-induced sleep loss compared with their controls (Fig 4L). These data indicate that starvation induces the release of AKH, and AKH/AKHR in the brain is very important for sleep regulation.

Because the starvation-induced sleep loss mainly started after the first LD shift, we tracked *akh* mRNA levels during starvation before and after conversion of light/dark. The results showed akh expression significantly increased only at ST15 (ZT3) after either the dark-light shift (Fig 4M) or light-dark shift (S5 Fig)—the time point at which starved flies suffered severe sleep loss. These results further confirmed the role of akh on starvation induced sleep loss.

In order to determine whether the role of AKH on starvation induced sleep loss is dependent or independent on development, we created *UAS-akh/+; tublin-Gal80ts/nsyb-Gal4* line and *tublin-Gal80ts/+; AKH-Gal4/UAS-AKH-RNAi* lines, the flies were cultured at 18°C and their sleeps at adulthood were monitored at 29°C under non-starvation and starvation conditions. Results showed that *akh* overexpression in the *nsyb* neurons (*UAS-akh/+; tublin-Gal80ts/nsyb-Gal4*) significantly increased starvation-induced sleep loss compared to the controls (*UAS-akh/+; tublin-Gal80ts/+* and *nsyb-Gal4/+*) (S6A Fig), while *akh* downregulated flies resisted to starvation induced sleep loss (S6B Fig). These results indicate that the role of AKH on starvation induced sleep loss is independent of development.

Finally, we created a rescue strain that drives *akh* expression with *AKHR-Gal4* in the *Akh*^*A*^ mutant flies. As expected, the rescued flies (*AKHR-Gal4/UAS-AKH*; *AkhA*) showed expanded s-LNv dorsal projections compared with the mutant flies with *AKHR-Gal4* (*AKHR-Gal4*; *AkhA*) or *UAS-akh* controls (*UAS-akh*; *AkhA*) (Fig 4N and 4O). Correspondingly, the mutant flies with rescued akh expression in the functional sites could rescue the phenotype of limited starvation-induced sleep loss in *Akh*^*A*^ mutant flies (Fig 4P). These results indicate AKH pathway is indispensable for starvation induced sleep loss, and it regulates starvation induced sleep loss through s-LNv dorsal projections remodeling.

### FOXO acts on the s-LNv dorsal projection remodeling as a downstream factor

**Fig 5:**
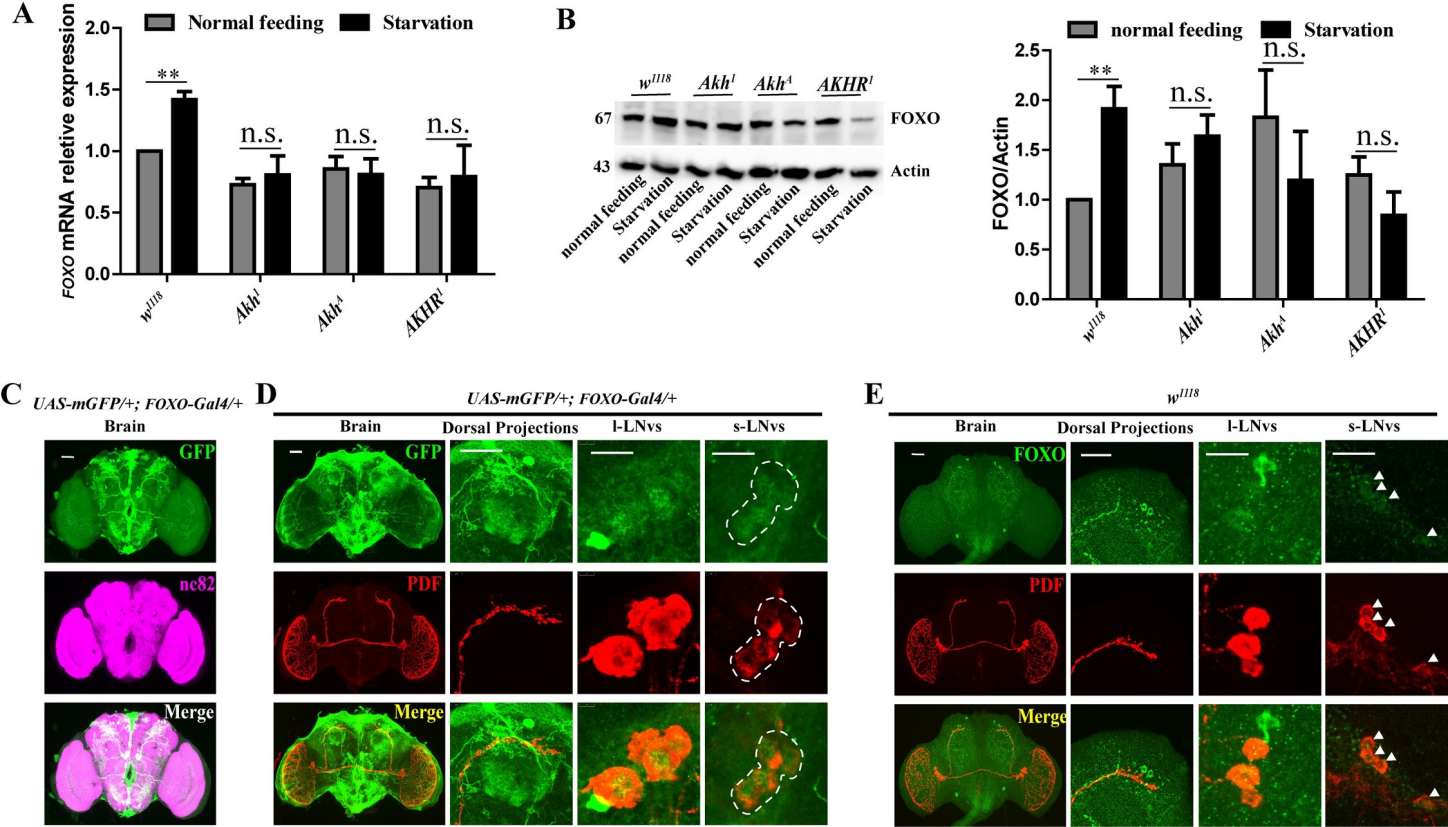
FOXO as starvation response factor acts downstream of AKH pathway, and was detected in the LNvs and in Surrounding s-LNv dorsal projections. (A) qRTPCR analysis of *foxo* amounts in *w^1118^*, *Akh^1^*, *Akh^A^*, and *AKHR^1^* flies under normal condition at ZT4 (gray column) and during starvation at ST16 (ZT4) (black column). Data were analyzed by t test, **p<0.001. (B) Activated FOXO levels in *w^1118^*, *Akh^1^*, *Akh^A^*, and *AKHR^1^* flies under normal condition at ZT4 and during starvation at ST16 (ZT4). The intensity of protein bands (B) was quantified by Image J and calculated as a relative value (the intensity of FOXO/the intensity of Actin) (C). Data were analyzed by t test, **p<0.001. (C) The brain immunofluorescence of *UAS-mGFP/+*; *foxo-Gal4/+* flies with anti-GFP (green) and anti-nc82 (magenta). (D) The immunofluorescence with anti-GFP (green) and anti-PDF (red) in *UAS-mGFP/+*; *foxo-Gal4/+* flies in brain, dorsal protocerebrum, l-LNvs, and s-LNvs. (E) The immunofluorescence with anti-FOXO (green) and anti-PDF (red) in *w^1118^* flies in brain, dorsal protocerebrum, l-LNvs, and s-LNvs.

In order to clarify the relationship between AKH and PDF-containing dorsal projections, we focused on the FOXO, which has been reported to act downstream of AKH as a transcription factor to mediate response to oxidation stress and starvation in *D*. *melanogaster* [5, 32]. By comparing the *foxo* mRNA levels in both *w*^*1118*^ and *akh* or *AKHR* mutant flies under non-starved and starved (ST16/ZT4) states, we found that the *foxo* mRNA levels in *w*^*1118*^ flies was significantly increased under starvation conditions, but did not in the *Akh*^*1*^, *Akh* ^*A*^ and *AKHR*^*1*^ flies (Fig 5A). We also detected unmodified FOXO protein levels in both *w*^*1118*^ and *akh* or *AKHR* mutant flies under non-starved and starved (ST16/ZT4) conditions using FOXO antibody, verified by the *foxo* downregulation driven by *foxo-gal4* (S7 Fig). The results showed similar increases like those detected for the *foxo* mRNA levels (Fig 5B). These results show that FOXO levels can be effectively blocked by elimination of AKH/AKHR signaling.

To confirm whether the FOXO is involved in the PDF-containing dorsal projection remodeling, we detected the expression location of *foxo* in the brain in the *UAS-mGFP/+*; *foxo-Gal4/+* flies. Results showed that the *foxo* has a wide range of expression in adult brain (Fig 5C). By further using the PDF antibody to mark the LNvs in the flies with mGFP driven by *foxo-Gal4*, we found that the *foxo*s were co- localized in the LNvs and the s-LNvs dorsal projections (Fig 5D). In another approach to co-localize the FOXO in *w*^*1118*^ flies using both PDF and FOXO antibodies, we showed that the FOXO were co-localized in the l-LNvs, s-LNvs and the s-LNv dorsal termini (Fig 5E).

**Fig 6:**
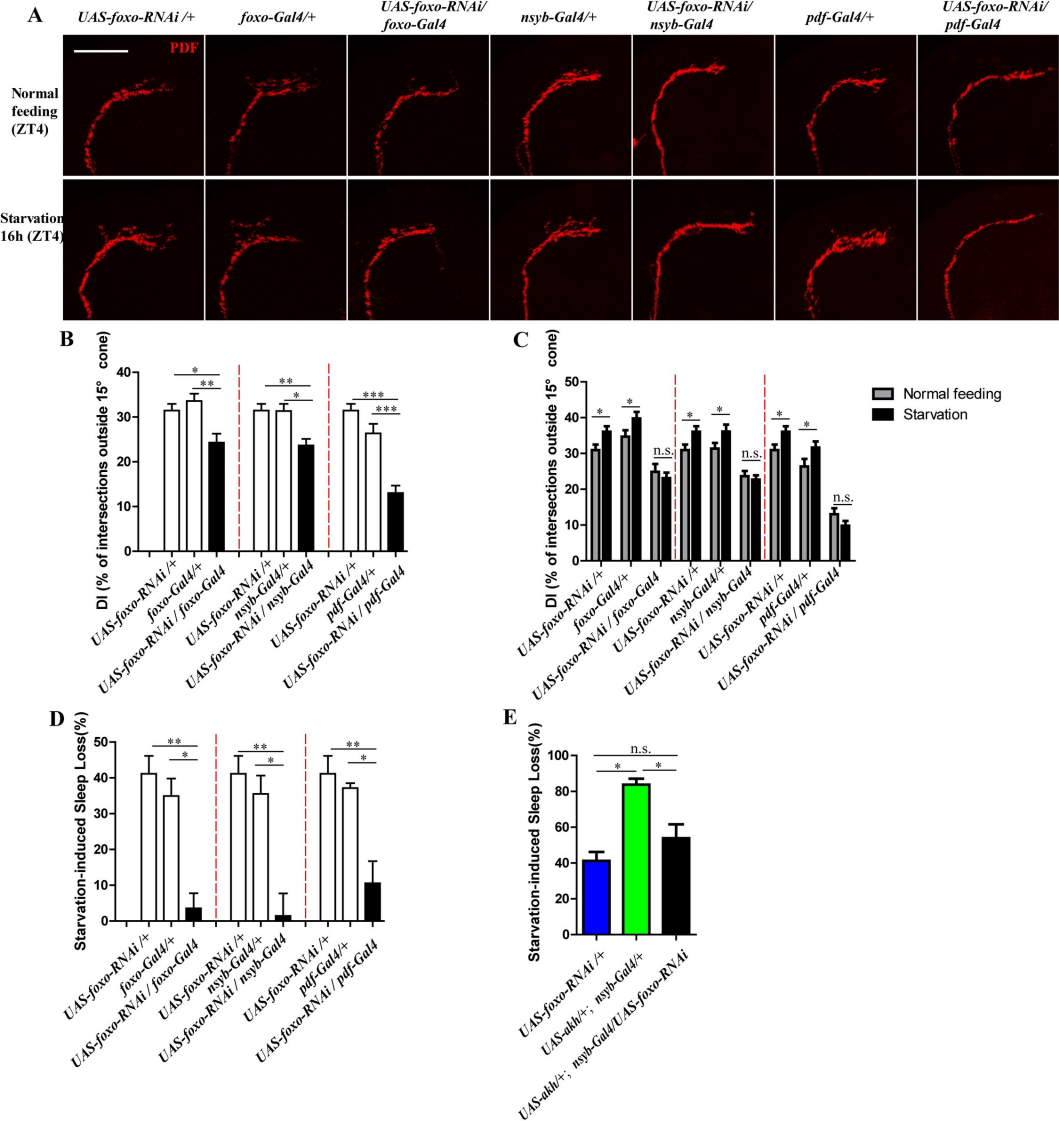
Downregulated *foxo* levels inhibit starvation-induced sleep loss and s-LNv dorsal projection openings. (A) PDF staining on s-LNvs dorsal projections in *foxo* down regulated flies and its control under normal feeding condition at ZT4 and starvation condition at ST16 (ZT4). The scale bar indicates 50um. (B) DIs of *foxo* down regulated flies (black column) and its controls (white column) under normal feeding condition at ZT4. Data were analyzed by One-way ANOVA, Tukey’s Multiple Comparison Test. * p<0.05, **p<0.001, *** p<0.0001. (C) DIs of *foxo* down regulated flies and its controls under normal condition at ZT4 (gray column) and during starvation at St16 (ZT4) (black column). Data were analyzed by t test, *p<0.05. (D) Starvation induced sleep loss in *foxo* down regulated flies (black column) and its controls (white column). Data were analyzed by One-way ANOVA, Tukey’s Multiple Comparison Test. *p< 0.05, **p<0.001. (E) Starvation induced sleep loss in *UAS-foxo-RNAi/+*, *UAS-akh/+*; *nsyb-Gal4/+* and *UAS-akh/+*; *nsyb-Gal4/UAS-foxo-RNAi* flies. Data were analyzed by One-way ANOVA, Tukey’s Multiple Comparison Test. *p< 0.05.

Additionally, we showed a role for FOXO on starvation induced PDF-containing dorsal projection remodeling. First, we down-regulated *foxo* by using a *UAS-foxo-RNAi* driven by *foxo*-gal4, *nsyb-Gal4* and *pdf-Gal4*, and then observed PDF-containing dorsal projections under non-starvation and starvation conditions. Under the non-starvation state, the *foxo* downregulation of flies in *foxo*, *nsyb* and *pdf* neurons significantly reduced DIs in the s-LNv dorsal termini compared with their controls (Fig 6A and 6B). However, they could resist starvation induced PDF-containing dorsal projections extension (Fig 6A and 6C). These data suggest that *foxo* is required in the starvation-induced s-LNvs dorsal projection increase. Subsequently, we monitored sleep behaviors under both states of starvation and non-starvation. Results showed that flies with the down-regulated *foxo* completely resisted the starvation-induced sleep loss compared to the same background of controls (Fig 6D). These results indicate that the down-regulation of *foxo* may effectively inhibit the starvation-induced sleep loss.

To further testify the relationship between AKH and FOXO on starvation induced sleep loss, we created the flies simultaneously overexpressing *akh* and downregulating *foxo* in *nsyb* neurons (*UAS-akh/+;nsyb-Gal4/UAS-foxo-RNAi*). The overexpressed *akh* flies had severe starvation induced sleep loss, which could be rescued by downregulated *foxo* (Fig 6E). Combining with the results from Fig 5A & 5B, these data suggests that FOXO acts downstream of AKH pathway.

### FOXO regulates starvation-induced sleep loss independent of development

**Fig 7:**
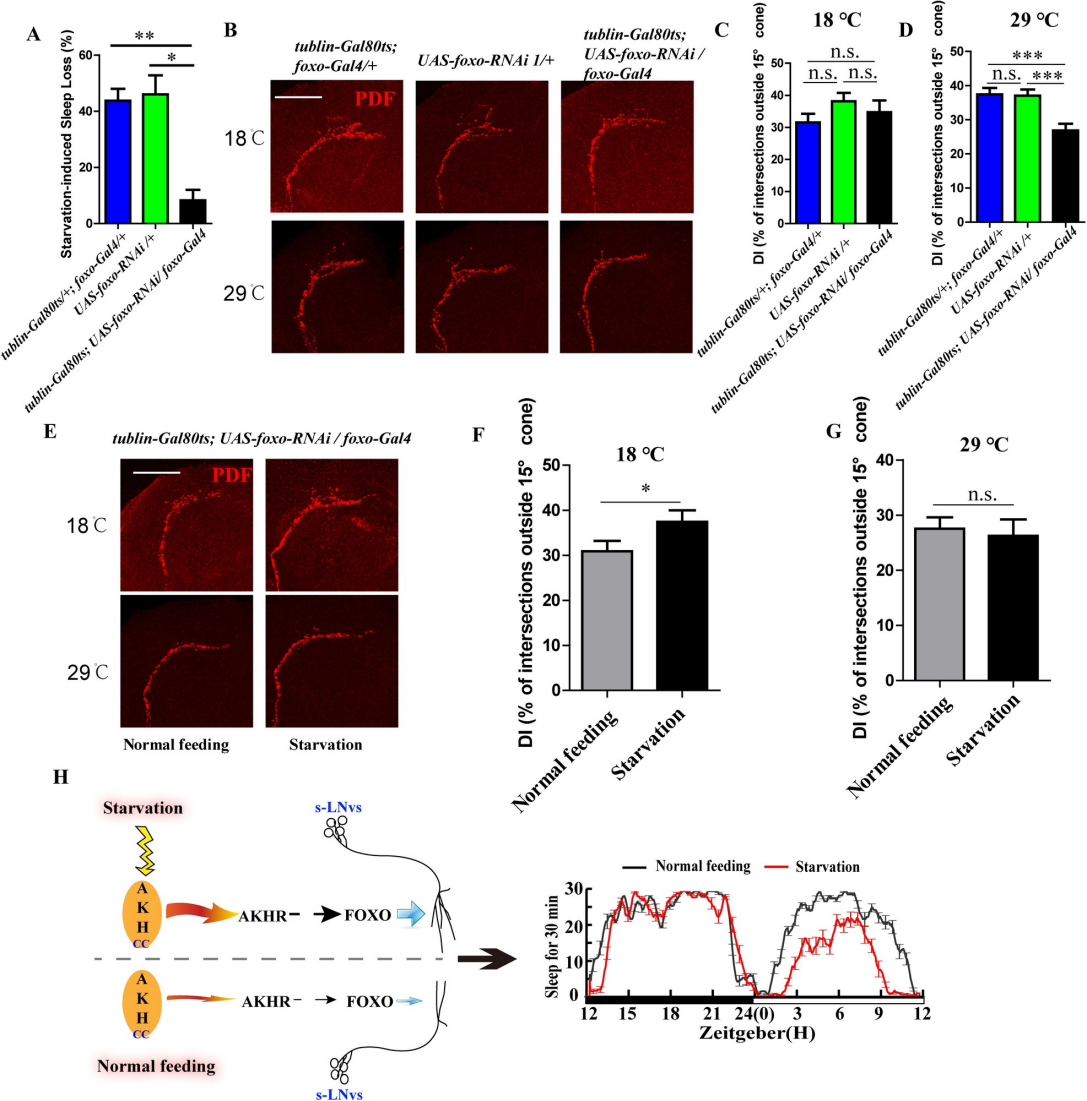
The roles of foxo on starvation-induced sleep loss and s-LNv dorsal projections are independent of development. (A) Starvation induced sleep loss in *tublin-Gal80ts/+*; *foxo-Gal4/+* (blue column), *UAS-foxo-RNAi/+* (green column), and *tublin-Gal80ts/+*; *UAS-foxo-RNAi/foxo-Gal4* (black column) flies. Data were analyzed by One-way ANOVA, Tukey’s Multiple Comparison Test, **p<0.001 and *p< 0.05. (B) PDF staining in *tublin-Gal80ts/+*; *foxo-Gal4/+*, *UAS-foxo-RNAi/+* and *tublin-Gal80ts/+*; *UAS-foxo-RNAi/foxo-Gal4* flies under normal feeding condition at ZT4 at 18°C and 29°C. The scale bar indicates 50um. (C and D) DIs of *tublin-Gal80ts/+*; *foxo-Gal4/+* (blue column), *UAS-foxo-RNAi/+* (green column), and *tublin-Gal80ts/+*; *UAS-foxo-RNAi/foxo-Gal4* (black column) flies under normal feeding condition at ZT4 at 18°C (C) and 29°C (D). Data were analyzed by One-way ANOVA, Tukey’s Multiple Comparison Test. ***p<0.0001. (E) PDF staining in *tublin-Gal80ts/+*; *UAS-foxo-RNAi/foxo-Gal4* flies under normal feeding condition at ZT4 and starvation condition at ST16 (ZT4) at 18°C and 29°C. The scale bar indicates 50um. (F and G) DIs of *tublin-Gal80ts/+*; *UAS-foxo-RNAi/foxo-Gal4* flies under normal feeding condition at ZT4 (gray column) and starvation condition at ST16 (ZT4) (black column) at 18°C (F) and 29°C (G). Data were analyzed by t test, *p< 0.05. (H) Working model of starvation induced sleep loss. AKH receives and transmits the starvation signal to the FOXO and then adjusts sleep through regulating the axonal remodeling of the s-LNv dorsal projections.

In order to determine whether the role of FOXO on starvation induced sleep loss or s-LNv dorsal projections is dependent or independent of development, we used foxo-gal4 to down regulate *foxo* expression only before sleep analysis by inhibiting its expression during larval stage via the temperature-sensitive tubulin-gal80 (*tublin-Gal80ts; foxo-Gal4*, *UAS-foxo-RNAi*), in which the tublin-gal80ts loses its inhibition to the foxo-gal4 at high temperature. Results showed that adult flies with down-regulated *foxo* (*tublin-Gal80ts;UAS-foxo-RNAi/foxo-Gal4*) significantly resisted starvation-induced sleep loss at 29°C compared to the controls (*tublin-Gal80ts;foxo-Gal4 and UAS-foxo-RNAi*) (Fig 7A), indicating that the role of FOXO on the starvation-induced sleep loss is independent of development. Moreover, we explored the s-LNvs dorsal projections in the same way like above. Results showed that the adult flies with the down-regulated *foxo* displayed closed s-LNv dorsal projection at 29°C but did not at 18°C (Fig 7B–7D). The flies with the down-regulated *foxo* after 16 h starvation increased PDF-containing dorsal projections at 18°C compared to those normal feeding flies (Fig 7E and 7F), but they did not show starvation induced s-LNv dorsal projections opening at 29°C (Fig 7E and 7G). These data demonstrate that the FOXO promotes defasciculation of the s-LNvs dorsal synapsis independent of development.

Taking together, our results show a regulatory pathway for starvation signal in flies, in which the AKH receives and transmits the starvation signal to FOXO and then adjusts sleep through regulating the axonal remodeling of the s-LNv dorsal projections (Fig 7H).

## Discussion

Previous studies have revealed that starvation-induced sleep loss occurs during ST12-24h, while the starvation-induced lipid utilization is already there after 5 h starvation [3]. It is worth noting that fruit flies are at the peak of activities during the moment of LD shift from lights on to lights off or vice versa, and are in a state of transition from wakefulness to sleep or vice versa. We found that starvation can be perceived as early as six hours after initiation of starvation, and starvation-induced sleep loss obviously starts after the first LD shift. Therefore, the first LD shift is very important for starvation-induced sleep loss. The starved flies exhibit longer sleep bout duration and smaller sleep bout number during the period before the first LD shift. The longer sleep bout duration accompanies a reduction of metabolic rate in flies [10], suggesting that sleep bout duration seems to be related to resistance to starvation. More importantly, the starvation-induced sleep loss after the first LD shift mainly is due to decrease of sleep bout number but not sleep bout duration, indicating that starvation is a stimulating factor maintaining awakeness and preventing sleep.

To determine why starved flies are not able to fall asleep, we turned to the pdf-expressing neurons (L-LNvs and s-LNvs) that have been reported as arousal neurons. Loss of PDF or PDF neurons increases sleep amount [19, 20]. Our results show that the PDF and PDF neurons play a role in starvation-induced sleep loss, in which starvation makes the s-LNv dorsal projections more divergent and seems to accelerate signal exchange between LNvs and other neurons, thereby inducing sleep loss.

The Blau’s lab has been working on starvation-induced sleep loss for many years. They showed that the mushroom bodies are dispensable for starvation-induced sleep loss, that the clk- expressing DN1s or LNds promote sleep during starvation, and that CLOCK (CLK) and CYCLE (CYC) limit starvation-induced sleep loss [7]. In the feedback loop of clock neurons for starvation-induced sleep loss, the LNvs as first responders promote arousal by activating DN1s, and the latter releases glutamate to inhibit the activity of pacemaker neurons, which are reported to control the balance of sleep-activity [18]. As the CLK/CYC activity increases, the excitability of DN1 is increased, further causing an inhibitory effect on the LNvs [33]. Thus, a homeostatic effect on sleep/awake in the starved flies seems to be produced because starvation causes sleep loss by inducing the s-LNv dorsal projection opening to transmit the wake-up signal to downstream neurons. This is a much stronger than the pathway of promoting sleep by the CLK/CYC-activated DN1s that inhibit the activity of awakening neurons.

AKH has been reported to have roles on starvation-induced metabolism shifting [5] and hyperactivity [6]. This study confirms the role of AKH on the starvation induced sleep loss and s-LNv dorsal projections remodeling. Loss of function of AKH or its receptor effectively inhibits starvation-induced divergence of the s-LNv dorsal projections and starvation-induced sleep loss. AKH, specifically expressed in CC, is proposed to regulate metabolic shifting through activating FOXO in the fat body when flies are hungry [5].

FOXO, acting downstream of AKH as a transcription factor, has been reported to mediate response to oxidation stress and starvation in *D*. *melanogaster* [5, 32]. The SIK3-HDAC4-FOXO axis is a significant constituent part in the AKH mediated starvation-induced metabolism shifting in the fat body [5], in which the SIK3 and HDAC4 are testified to impact Drosophila circadian behavior and the male sex-driving rhythm by modulating the DN1 clock neurons [34]. Additionally, FOXO also regulates feeding behavior and food intake by regulating sNPF/NPY expression from the fasting to feeding transition in Drosophila and mammals [35], and it impacts synapse plasticity at the neuromuscular junctions (NMJs) through a downstream target—the mitotic kinesin MKLP1/Pavarotti (Pav-KLP) in the larval flies [36, 37]. In adult flies, FOXO regulates diet-induced synaptic plasticity in the CM9 motoneuron through eif-4e binding protein (4eBP)-dependent complexin [38]. In this study, FOXO is shown to be very important for regulation of the starvation-induced sleep loss. First, FOXO is shown to be downstream of AKH/AKHR in the starvation signal, since loss of function of AKH or AKHR blocks the activation of FOXO induced by starvation. Second, FOXO exists in the s-LNvs cell soma and its dorsal projections, and its down regulation inhibits the starvation-induced sleep loss and starvation-induced s-LNv dorsal projection remodeling.

Some factors has been reported to regulate remodeling of the s-LNv dorsal projections, in which the Mef2 is directly regulated by CLK/CYC to cause morphological changes in s-LNv projections through some genes functioning in neuronal remodeling, such as the Fasciclin 2 (Fas2) [27], metalloproteinases1 (Mmp1) and ecdysone receptor [39]. MiR-92a regulated by light and circadian rhythm impacts the s-LNv dorsal projection remodeling through silent information regulator 2 (sirt2) [28]. Sirt2 preventing the PTEN-induced kinase 1 (PINK1) induces mitochondrial dysfunction and loss of the dopaminergic neuron via FOXO [40]. Mef2 and FOXO, regulated by HDAC4, are widespread transcription factors that regulate transcription of the same genes in Drosophila [41]. These findings suggest that the s-LNv dorsal projections are co-regulated by the circadian rhythm-related Mef2 and environment factors related to sirt2 and FOXO.

Under the normal condition, sleep is regulated by circadian rhythm and sleep homeostasis. However, the organisms always face different kinds of challenges in the surrounding environment, which should lead to corresponding change of sleep-wake state to meet the needs of survival. Food inanition is a common survival challenge for organisms, which has great influence on behaviors. This study revealed the starvation signal is important for sleep regulation of Drosophila through AKH-FOXO mediated s-LNv dorsal projections remodeling.

## Materials and methods

### Animal breeding and maintenance

The *Pdf*^*01*^ (stock No. 26654), *Pdfr*^*5304*^ (stock No. 33068), *pdf-Gal4* (*R61G12-Gal4*, stock No. 41286), *UAS-CD8*::*GFP* (stock No. 5137), *UAS-akh* (stock No. 27343), *AKH-Gal4* (stock No. 25684), *nsyb-Gal4* (stock No. 51941), *foxo-Gal4* (stock No. 112303), *UAS-FOXO-RNAi* (stock No. 27656) were purchased from the Bloomington Drosophila Stock Center (Indiana University). The *foxo-Gal4* (stock No. 112303) were purchased from *Kyoto* Drosophila Stock Center. *UAS-rpr* were from the laboratory of Dr. Yi Rao (Chinese Institute for Brain Research, China). The *AKHR-Gal4* were from the laboratory of Dr. Liming Wang (Zhejiang University, China). The *AKHR*^*1*^ and *Akh*^*A*^ were from the laboratory of Dr. Ronald P. Kühnlein (Max Planck Institute for Biophysical Chemistry, Germany). They have been previously described [4, 31]. *Akh*^*1*^ was from the laboratory of Dr. Daniela Hlavkova (Biology Centre CAS Institute of Entomology, Czech Republic) and has been previously described [30]. All the flies used were crossed into a w1118 background.

Flies were reared on a standard normal feeding diet at 25°C and 65% relative humidity in a 12 hr L:12 hr D (LD) cycle. Experimental flies were collected after emergence and transferred to a standard incubator for 2–3 days with normal feeding conditions. Hybrid strains bearing tublin-Gal80 were kept, collected and transferred for adaption in an 18°C incubator. The normal feeding diet containing 8g Agar, 31.62g Sucrose, 63.2g Glucose, 77.7g Maize meal, 32.2g yeast, 0.726g Cacl2 per litre of distilled water. The diet for food deprivation was 1% agar.

### Sleep behavioral assays

Three- to five-day-old flies were housed in monitor tubes (5[W] × 65[L] mm) with fly food for one-two days for adaption and then changed to the same type of tubes with normal fly food or 1% agar diet at the specified time. Experiments were performed in an incubator at a temperature of 25 ± 1°C and a relative humidity of 65%. Light was turned on at ZT0 (local time 06:30) and off at ZT12 (local time 18:30). The sleep activity was recorded using the Drosophila Activity Monitoring System (Trikinetics, Waltham, MA). The data were scanned by DAM, and analyzed by pySolo software.

### Immunofluorescence

Brains from 3-day-old adult flies were fixed by immersion in ice cold 4% paraformaldehyde in PBS at room temperature for 2 h or 4°C for 12h, dissected in chilled phosphate buffered saline (PBS, pH 7.4), and then rinsed three times in PBS with 0.5% Triton X-100 (PBST) for 15 min each. Brains were first incubated overnight at 4°C with primary antibodies (mouse anti-PDF 1:300, mouse anti-nc82 1:300, Rabbit anti-FOXO 1:200, Rabbit anti-GFP 1:1000) after blocking 1 h in PBS with 10% goat serum (PNT). They were then washed with PBST 3 times for 15 min each. Then the corresponding second antibody (Goat Anti-Rabbit DyLight 488 1:300, Goat Anti-Mouse Cy5 1:300) was incubated with the brains three hours at room temperature, which were then washed three times with PBST at room temperature. Finally, the brains were sealed on a slide with a small slot with sealed buffer. The nc82 antibody were purchased from DSHB. Images were acquired as a z stack with Leica SP8 confocal microscope equipped with the Fluoview software LAS X (Leica). The s-LNvs dorsal termini defasciculation indexes (DI) were calculated based on modified sholl’s method [27]. Fifteen concentric circles spaced 10 mm apart in sequence and a 15° cone from the center of circles were set up as computing baseboard, s-LNv dorsal projections in each brain hemisphere were placed on the baseboard with the point where dorsal ramification opens on the center of circle, and made the most intersections in the 15° cone. The number of intersections between axon branches and the concentric circles were noted down. The DI defined as the ratio of intersections outside the 15° cone to the total number of intersections. The Quantitative analysis was performed using ImageJ (https://imagej.en.softonic.com/)

### Quantitative real-time PCR

3-day-old male flies at a specific time were sampled and grinded with Trizol Reagent (TIANGEN) according to the manufacturer’s protocol. These then went through treatment with chloroform for removing protein impurity, isopropyl alcohol for precipitating nucleic acid, 75% ethanol for washing, and Rnase-free water for dissolving the precipitate. The concentration of the nucleic acid mixture was measured, and genomic DNA was removed and the mRNA was reversed transcribed using PrimeScript RT Reagent Kit with gDNA Eraser (TaKaRa). The Quantitative real-time PCR assay was performed using an Applied Biosystem Step One Real Time PCR system (Applied Biosystem, Foster, CA, USA) and SuperReal PreMix Plus (SYBR Green) (TIANGEN). The primers for amplifying: *dAkh*: For (5′- ATGAATCCCAAGAGCGAAGT -3′) and Rev (5′- CTACTCGCGGTGCTTGCAGTCCAGA -3′); *foxo*: For (5′- TTCTACCCCATGATGGACGG -3′) and Rev (5′- GCATTCGCATTCTGTATAGCCT -3′); *actin*: For (5′- CAGAGCAAGCGTGGTA TCCT -3′) and Rev (5′- CTCATTGTAGAAGGTGTGGTGC -3′).

### Western blot

The heads of the experimental flies were collected at a specific time for protein extraction, and each sample contained sixty heads. The heads were homogenized in 120ul RIPA lysis buffer (CWBIO) with protease inhibitor by a tissue grinder, and then the tissue homogenate was centrifuged for ten minutes in 13000rpm at 4°C. The supernatant was taken for protein detection. An appropriate amount of supernatant with loading buffer was boiled for seven minutes and then loaded onto a prepared 10% polyacrylamide gel for protein separation at 80 V for a half-hour and 120V for two hours in electrophoretic buffer. Then the separated protein was transferred from gel to PVDF membrane at 80 V for two hours in membrane transfer buffer. The membrane was washed in PBST for 15min and incubated in 5% skim milk in PBST for one hour at room temperature. The primary antibody (Rabbit anti-FOXO 1:1000, Mouse anti-Actin 1:2000) in PBST containing 5% skim milk was used to incubate the membrane over night at 4°C. After three 15min washes in PBST, the membrane was incubated in a second antibody (Goat Anti-Rabbit IgG- HRP 1:1000, Goat Anti-Mouse IgG-HRP 1:2000) in PBST containing 5% skim milk for two hours. The membrane was washed and then visualized using C600 multifunctional molecular imaging system (Azure). Fluorescent intensity was measured using the Image J software.

### Statistics

Statistical analyses were performed using GraphPad Prism7 (GraphPad Software RRID: SCR_00 2798), specific comparison methods are marked in the corresponding figure annotation, asterisks were used for indicating the statistically significant differences, *** indicate p<0.0001, ** indicate p<0.001, *indicate p< 0.05, n.s. indicated no significant difference.

## Supporting information

S1 Fig Starvation had no influence on PDF content. (A) The immunostaining of *UAS-CD8*::*GFP/+; pdf-Gal4/+* flies under normal feeding condition at ZT4 and starvation condition at ST16 (ZT4) with anti-PDF (red). The scale bar indicates 50um. (B-C) The immunofluorescence intensity of PDF in s-LNv dorsal projections (B) and LNv somas (C) under normal condition at ZT4 and during starvation at ST16 (ZT4) in *UAS-CD8*::*GFP/+; pdf-Gal4/+* flies. The immunofluorescence intensities were quantified by Image J. The data were analyzed by t test, *** indicate p<0.0001, ** indicate p<0.001, *indicate p< 0.05.
(TIF)

S2 Fig *akh* specifically expressed in CC. The GFP staining of UAS-CD8::GFP/+; AKH-Gal4 in the CC. The scale bar indicates 100um.
(TIF)

S3 Fig The expression pattern of *AKHR-Gal4*, *nsyb-Gal4 and ppl-Gal4*. **** (A) The brain immunofluorescence of *AKHR-Gal4/ UAS-CD8*::*GFP* flies with anti-GFP (green). (B) The fat body immunofluorescence of *AKHR-Gal4/ UAS-nGFP* flies with anti-GFP (green) and anti-DAPI (blue). (C) The brain immunofluorescence of *UAS-CD8*::*GFP/+; nsyb-Gal4/+* flies with anti-GFP (green). (D) The fatbody immunofluorescence of *UAS-nGFP/+; nsyb-Gal4/+* flies with anti-GFP (green) and anti-DAPI (blue). (E) The brain immunofluorescence of *ppl-Gal4/ UAS-CD8*::*GFP* flies with anti-GFP (green). (F) The fatbody immunofluorescence of *ppl-Gal4/UAS-nGFP* flies with anti-GFP (green) and anti-DAPI (blue). All scale bars indicate 50um.
(TIF)

S4 Fig Trehalose level in akh overexpression flies. Trehalose level in *akh* overexpression flies (black column) and its controls (white column). Data were analyzed by One-way ANOVA, Tukey’s Multiple Comparison Test. *p<0.05, **p<0.001.
(TIF)

S5 Fig qRT-PCR analysis of *akh* level. qRT-PCR analysis of *akh* amounts in *w*^*1118*^ flies during starvation before and after first light to dark shift. Black cube indicates *akh* expression in the normal feeding condition, and the red triangle indicates the akh expression in the starvation condition (relative to its expression in the normal feeding). Data were analyzed by t test, *p< 0.05.
(TIF)

S6 Fig Role of AKH on starvation induced sleep loss is independent of development. (A) Starvation induced sleep loss in *nsyb-Gal4/+* (blue column), *UAS-AKH/+; tublin-Gal80ts/+* (green column), and *UAS-AKH/+; tublin-Gal80ts/nsyb-Gal4* (black column) flies. Data were analyzed by One-way ANOVA, Tukey’s Multiple Comparison Test, ***p<0.0001. (B) Starvation induced sleep loss in *tublin-Gal80ts/+; AKH-Gal4/+* (blue column), *UAS-AKH-RNAi/+* (green column), and *tublin-Gal80ts/+; AKH-Gal4/ UAS-AKH-RNAi* (black column) flies. Data were analyzed by One-way ANOVA, Tukey’s Multiple Comparison Test, *p<0.05.
(TIF)

S7 Fig Verification of FOXO antibody and *foxo-Gal4*. (A and B) Activated FOXO levels in *foxo-Gal4/+*, *UAS-foxo-RNAi/+*, and *UAS-foxo-RNAi/foxo- Gal4* flies. The intensity of protein bands (A) was quantified by Image J and calculated as a relative value (the intensity of activated FOXO/the intensity of Actin) (B). Data were analyzed by One-way ANOVA, Tukey’s Multiple Comparison Test. *** p<0.0001. (C) The fat body immunofluorescence of *UAS-nGFP/+; foxo-Gal4/+* flies with anti-GFP (green), anti-FOXO (red) and anti-DAPI (blue). (D) The brain immunofluorescence of *UAS-CD8*::*GFP/+; foxo-Gal4/+* flies with anti-GFP (green) and anti-FOXO (red). (E) The pars intercerebralis, dorsal, dorsal protocerebrum and ventral lateral part of brain immunofluorescence of *UAS-CD8*::*GFP/+; foxo-Gal4/+* flies with anti-GFP (green) and anti-FOXO (red). All scale bars indicate 50um.
(TIF)
