# Supplementary figures and images for: AKH-FOXO pathway regulates starvation-induced sleep loss through remodeling of the small ventral lateral neuron dorsal projections
Source: PLoS Genet. 2020 Oct 26;16(10):e1009181. doi: 10.1371/journal.pgen.1009181 (PMC7644095; doi:10.1371/journal.pgen.1009181)

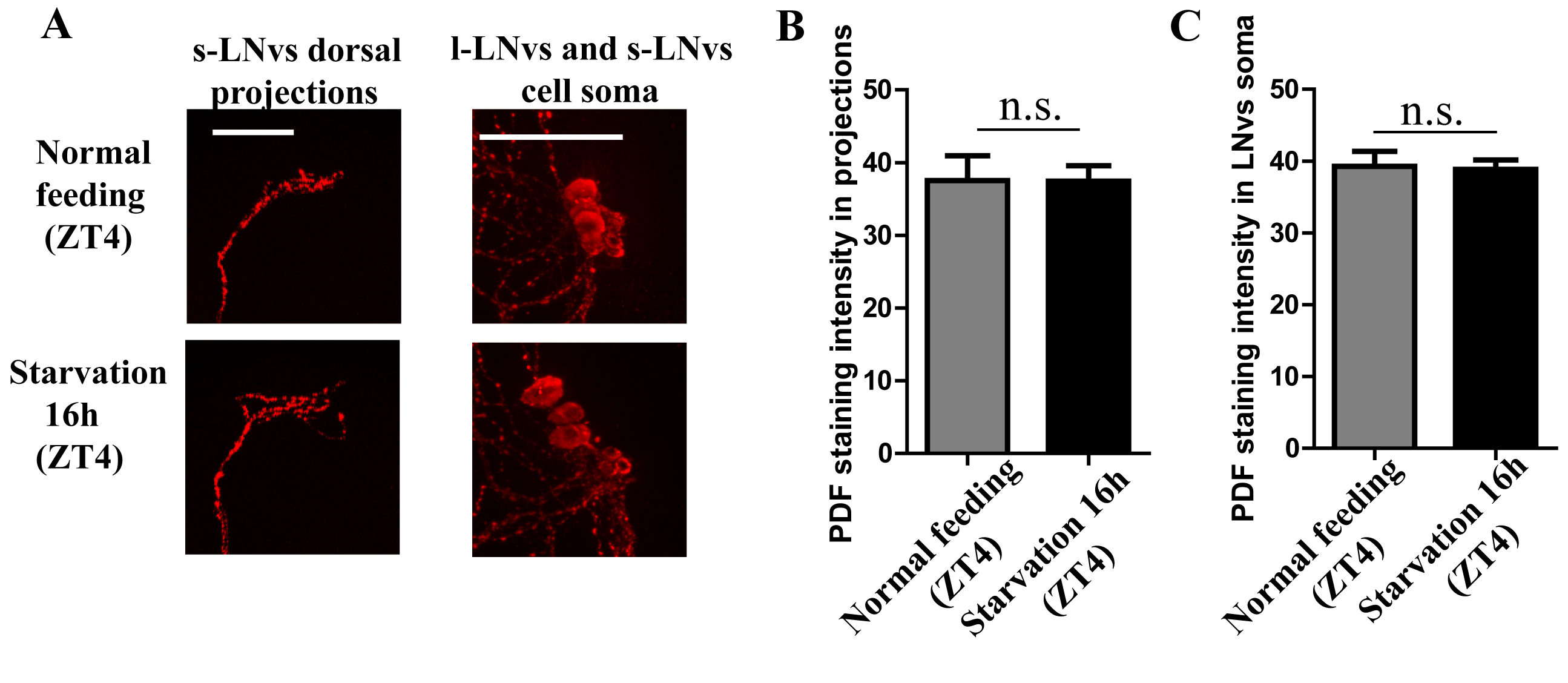

Supplement: S1 Fig — (A) The immunostaining of UAS-CD8::GFP/+; pdf-Gal4/+ flies under normal feeding condition at ZT4 and starvation condition at ST16 (ZT4) with anti-PDF (red). The scale bar indicates 50um. (B-C) The immunofluorescence intensity of PDF in s-LNv dorsal projections (B) and LNv somas (C) under normal condition at ZT4 and during starvation at ST16 (ZT4) in UAS-CD8::GFP/+; pdf-Gal4/+ flies. The immunofluorescence intensities were quantified by Image J. The data were analyzed by t test, *** indicate p<0.0001, ** indicate p<0.001, *indicate p< 0.05. (TIF) [file pgen.1009181.s001.tif]

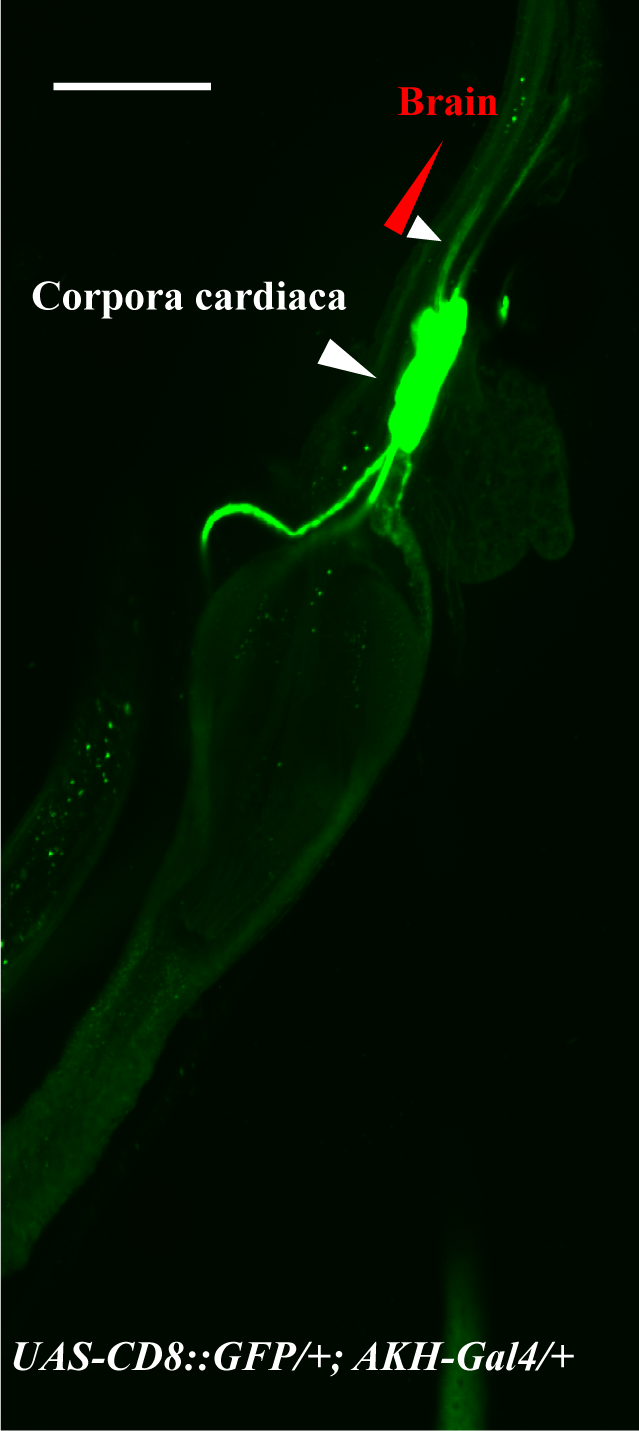

Supplement: S2 Fig — The GFP staining of UAS-CD8::GFP/+; AKH-Gal4 in the CC. The scale bar indicates 100um. (TIF) [file pgen.1009181.s002.tif]

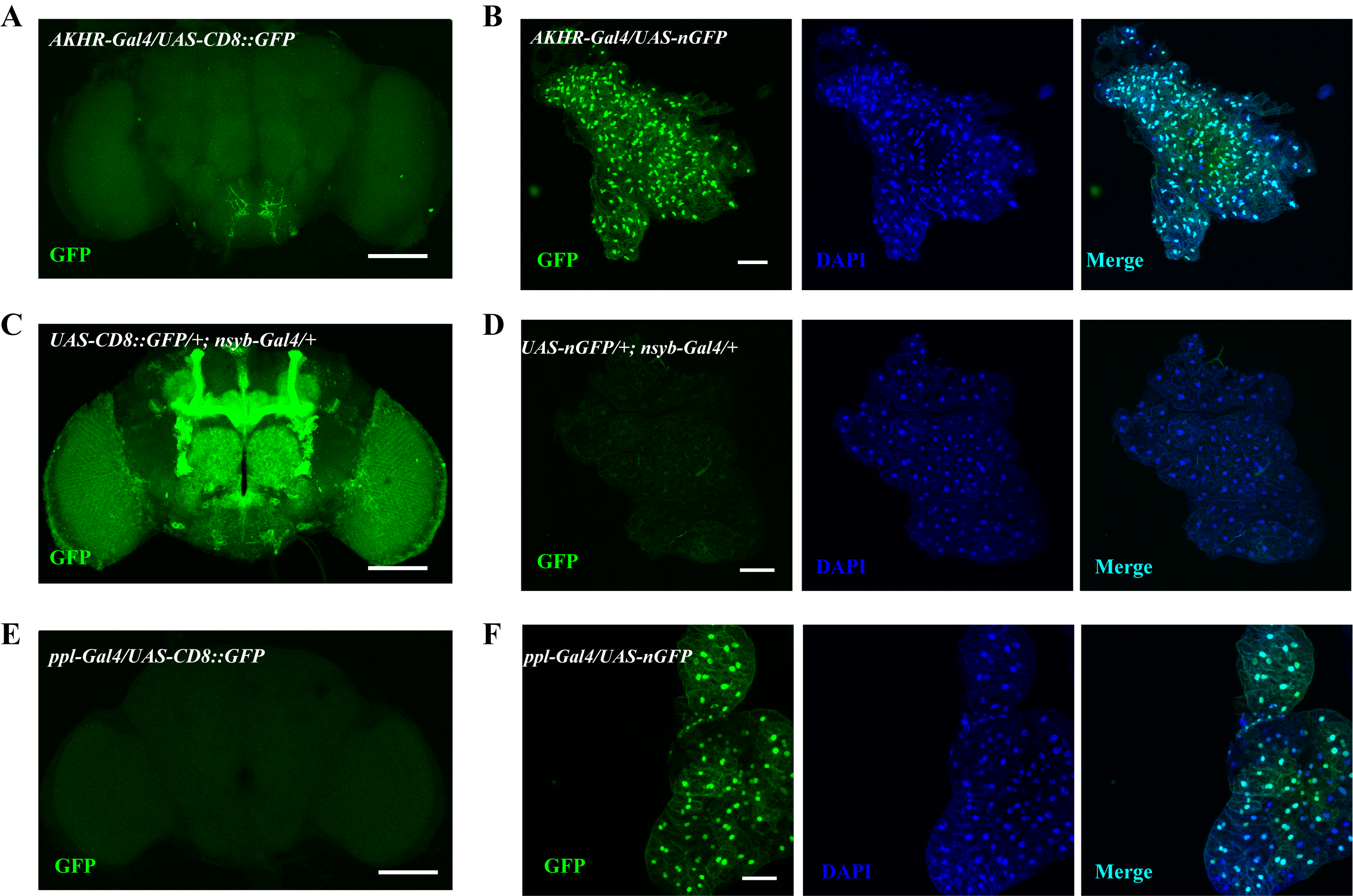

Supplement: S3 Fig — (A) The brain immunofluorescence of AKHR-Gal4/ UAS-CD8::GFP flies with anti-GFP (green). (B) The fat body immunofluorescence of AKHR-Gal4/ UAS-nGFP flies with anti-GFP (green) and anti-DAPI (blue). (C) The brain immunofluorescence of UAS-CD8::GFP/+; nsyb-Gal4/+ flies with anti-GFP (green). (D) The fatbody immunofluorescence of UAS-nGFP/+; nsyb-Gal4/+ flies with anti-GFP (green) and anti-DAPI (blue). (E) The brain immunofluorescence of ppl-Gal4/ UAS-CD8::GFP flies with anti-GFP (green). (F) The fatbody immunofluorescence of ppl-Gal4/UAS-nGFP flies with anti-GFP (green) and anti-DAPI (blue). All scale bars indicate 50um. (TIF) [file pgen.1009181.s003.tif]

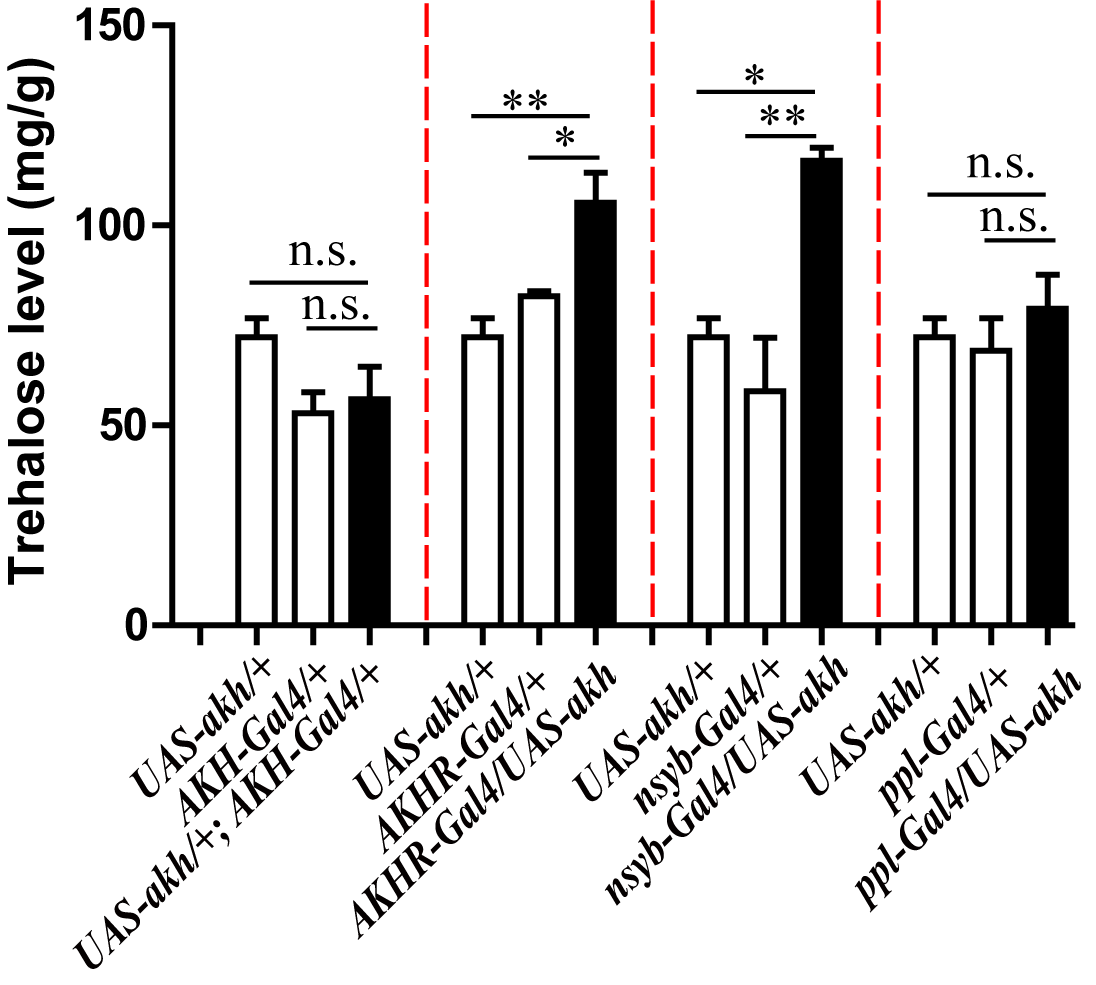

Supplement: S4 Fig — Trehalose level in akh overexpression flies (black column) and its controls (white column). Data were analyzed by One-way ANOVA, Tukey’s Multiple Comparison Test. *p<0.05, **p<0.001. (TIF) [file pgen.1009181.s004.tif]

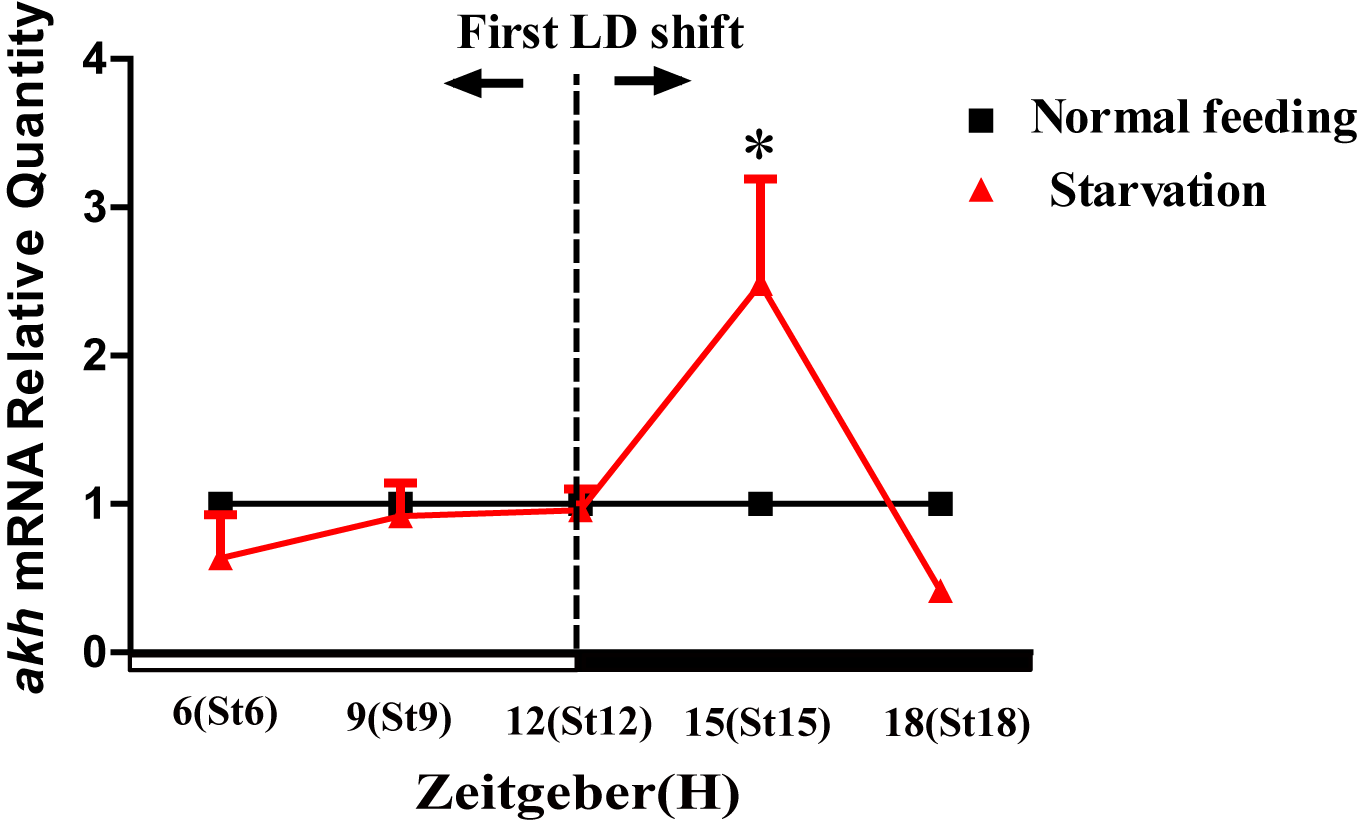

Supplement: S5 Fig — qRT-PCR analysis of akh amounts in w1118 flies during starvation before and after first light to dark shift. Black cube indicates akh expression in the normal feeding condition, and the red triangle indicates the akh expression in the starvation condition (relative to its expression in the normal feeding). Data were analyzed by t test, *p< 0.05. (TIF) [file pgen.1009181.s005.tif]

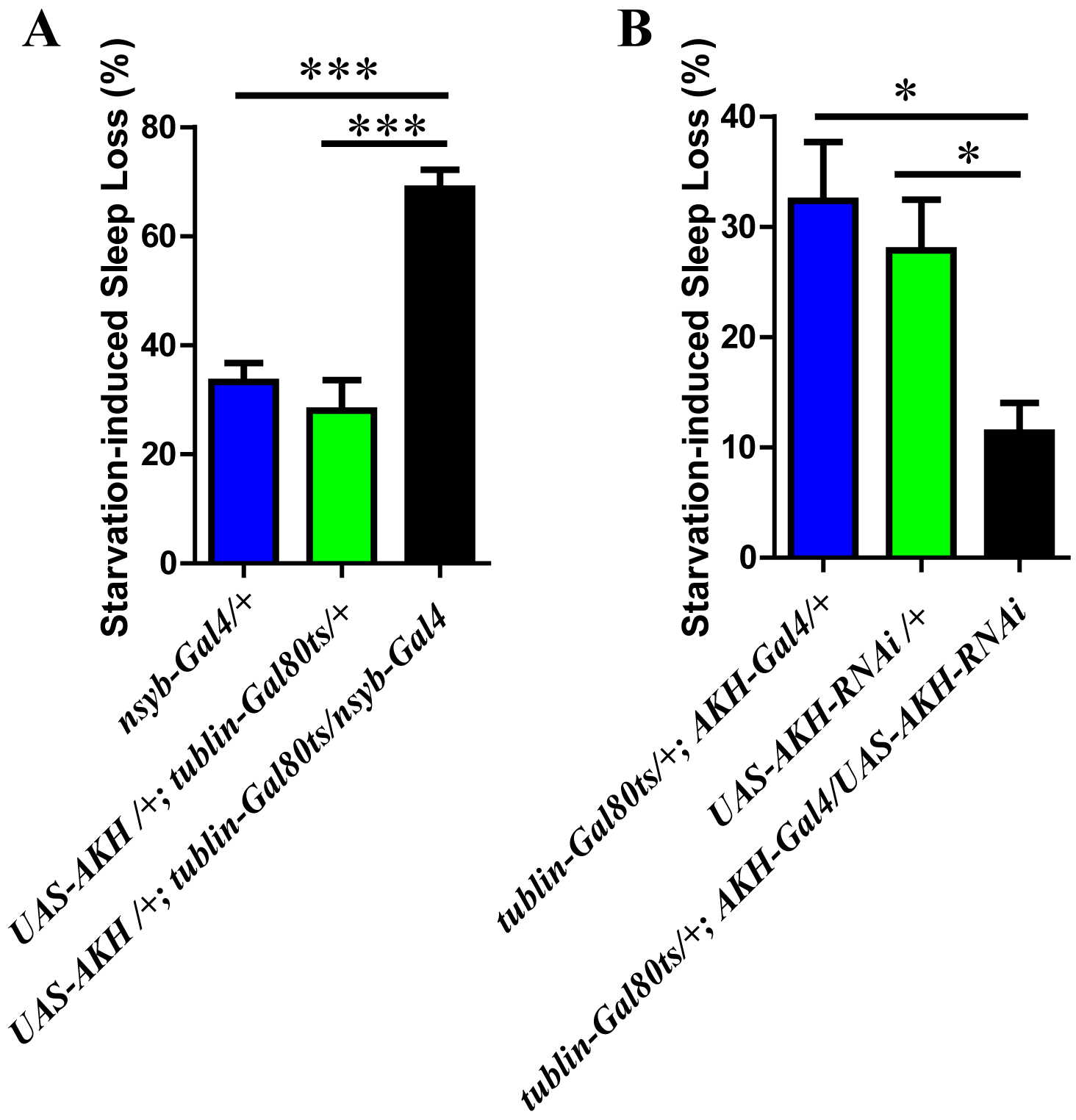

Supplement: S6 Fig — (A) Starvation induced sleep loss in nsyb-Gal4/+ (blue column), UAS-AKH/+; tublin-Gal80ts/+ (green column), and UAS-AKH/+; tublin-Gal80ts/nsyb-Gal4 (black column) flies. Data were analyzed by One-way ANOVA, Tukey’s Multiple Comparison Test, ***p<0.0001. (B) Starvation induced sleep loss in tublin-Gal80ts/+; AKH-Gal4/+ (blue column), UAS-AKH-RNAi/+ (green column), and tublin-Gal80ts/+; AKH-Gal4/ UAS-AKH-RNAi (black column) flies. Data were analyzed by One-way ANOVA, Tukey’s Multiple Comparison Test, *p<0.05. (TIF) [file pgen.1009181.s006.tif]

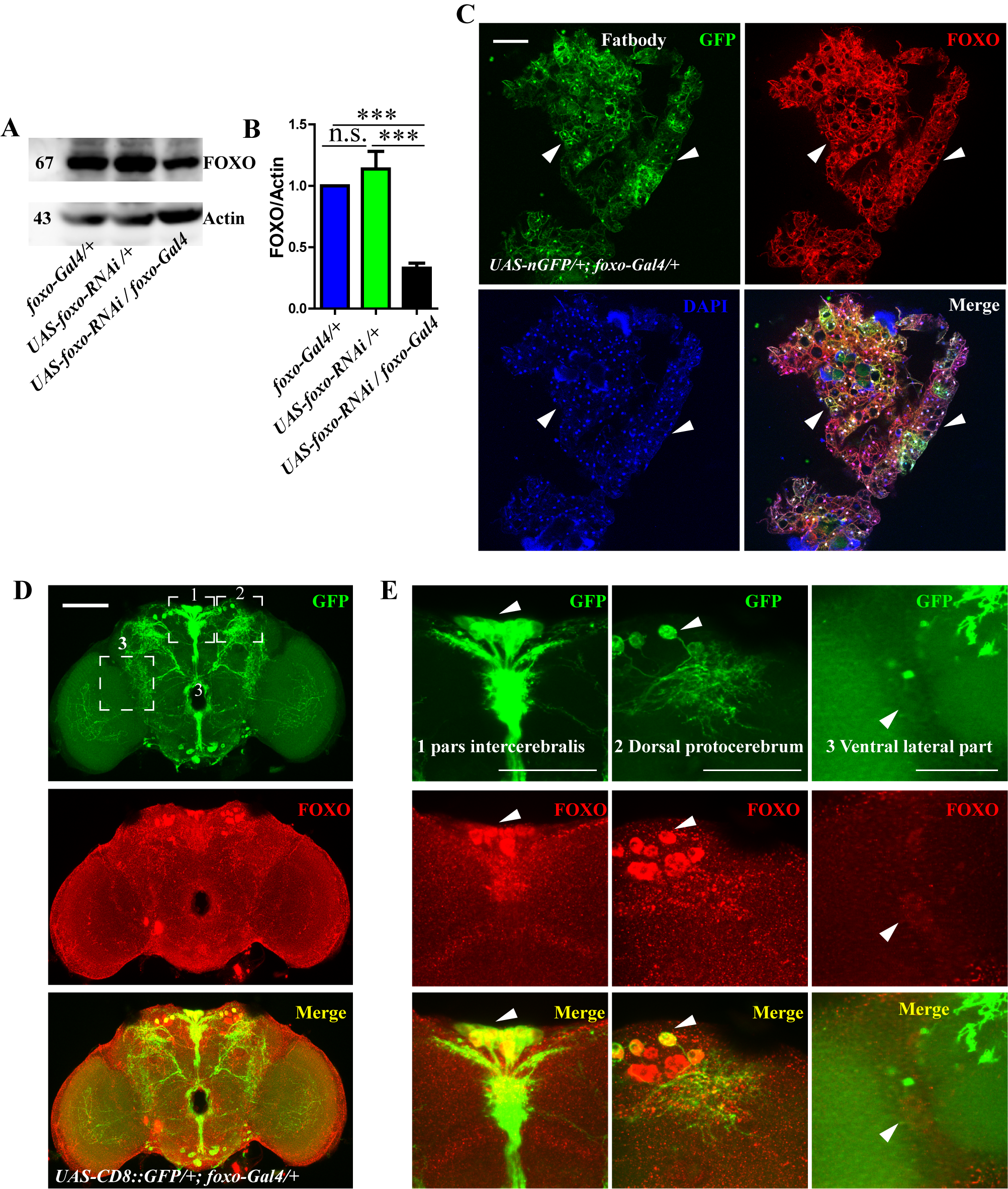

Supplement: S7 Fig — (A and B) Activated FOXO levels in foxo-Gal4/+, UAS-foxo-RNAi/+, and UAS-foxo-RNAi/foxo- Gal4 flies. The intensity of protein bands (A) was quantified by Image J and calculated as a relative value (the intensity of activated FOXO/the intensity of Actin) (B). Data were analyzed by One-way ANOVA, Tukey’s Multiple Comparison Test. *** p<0.0001. (C) The fat body immunofluorescence of UAS-nGFP/+; foxo-Gal4/+ flies with anti-GFP (green), anti-FOXO (red) and anti-DAPI (blue). (D) The brain immunofluorescence of UAS-CD8::GFP/+; foxo-Gal4/+ flies with anti-GFP (green) and anti-FOXO (red). (E) The pars intercerebralis, dorsal, dorsal protocerebrum and ventral lateral part of brain immunofluorescence of UAS-CD8::GFP/+; foxo-Gal4/+ flies with anti-GFP (green) and anti-FOXO (red). All scale bars indicate 50um. (TIF) [file pgen.1009181.s007.tif]
